# Synaptojanin1 regulates synaptic dopamine release and axonal integrity via retromer-dependent endosomal sorting

**DOI:** 10.21203/rs.3.rs-9695405/v1

**Published:** 2026-06-18

**Authors:** Nirmal Kumar, Elnaz Khezerlou, Jacqueline Saenz, Justin Cai, Hanna Caiola, Shlok Nayak, Eden Lee, Sanjana Surya Prakash, Ulrik Gether, Huaye Zhang, Ping-Yue Pan

**Affiliations:** 1 Department of Neuroscience and Cell Biology, Rutgers University Robert Wood Johnson Medical School, 675 Hoes Lane West, Piscataway, NJ 08854, USA; 2 Graduate Program in Neuroscience, Rutgers University; 3 Master Program in Biomedical Sciences, Rutgers University; 3 Department of Neuroscience, University of Copenhagen, Blegdamsvej 3, DK-2200 Copenhagen, Denmark

## Abstract

Synaptic dysfunction is increasingly recognized as an early feature of Parkinson’s disease (PD); however, synaptic mechanisms contributing to early dopamine release defects and neurodegeneration remains poorly understood. Here we identify a presynaptic endosomal-dependent mechanism supporting dopamine release and axonal integrity. Loss of the PD-associated lipid enzyme Synaptojanin1 impairs dopamine release due to endosomal retention of the dopamine D2 autoreceptor and dopamine transporter (DAT). Conditional deletion of Synaptojanin1 in mouse dopamine neurons results in endosomal swelling within striatal DAT clusters and PD-like locomotor deficits. Mechanistically, Synaptojanin1 remodels endosomal phosphatidylinositol 4-phosphate to facilitate the recruitment of the PD-associated retromer component VPS35. Notably, overexpressing VPS35 rescues presynaptic sorting defects in Synaptojanin1-deficient dopamine neurons despite lipid impairments. Furthermore, Synaptojanin1 and VPS35 exhibit correlated expression and dopamine-induced co-clustering in axons, supporting their broader roles in regulating synaptic surface proteins. Our work demonstrates a lipid-dependent endosomal mechanism that may contribute to motor deficits in early PD.

## Teaser

Presynaptic endosomes can be hijacked by two Parkinson’s disease genes to impact synaptic integrity and motor function.

## Introduction

Parkinson’s disease (PD) is a second most common neurodegenerative disorder primarily affecting brain dopamine and movement. While the precise cause of PD remains unclear, increasing evidence points to synaptic dysfunction as an early pathological event prior to neurodegeneration^1–6^. The most recent genome-wide association study of over 63,000 PD cases further solidifies neuronal processes and presynaptic terminals as vulnerable subcellular compartments^3,4^. However, our understanding of presynaptic mechanisms that support neuronal signaling and axonal maintenance remains incomplete^7–10^. There is a growing interest in autophagy as an essential mechanism for maintaining synaptic proteostasis^10^, whereas other pathways are less understood. A recent study of PD-linked Auxilin (*DNAJC6* or PARK19^11^) knockout mice identified severe defects in the protein composition of synaptic vesicles, suggesting not only protein level but its localization also mattered and may contribute to synaptic defects and motor impairments^12^. Sorting and localization of membrane cargo proteins is carried out by endosomes and the VPS35 retromer complex^13–18^. Significant advances have been made in understanding endosomal sorting and signaling pathways, yet how these processes operate at presynaptic terminals remains largely unknown. Evidence from the Auxilin model suggests an underappreciated role of synaptic endocytic genes in presynaptic sorting and highlights the need to elucidate endosomal sorting machineries in the presynaptic terminal, particularly dopaminergic terminals.

At presynaptic terminals, Auxilin cooperates with Synaptojanin1 (*SYNJ1*, also known as PARK20^19–23^), a Parkinsonism-associated lipid phosphatase, to remove the Clathrin coats from endocytic vesicles. Recent studies have shown additive effects of Auxilin and Synaptojanin1 (Synj1) in dopaminergic axonal dystrophy, and that Synj1 can reverse lipid defects and neurodegeneration in Auxilin mutant flies^24,25^. Synj1 is a presynaptically enriched phosphoinositide phosphatase with two enzymatic domains^26^. The SAC1-like domain hydrolyzes phosphatidylinositol 3-phosphate (PI3P), phosphatidylinositol 4-phosphate (PI4P) and phosphatidylinositol (3,5)-bisphosphate (PI(3,5)P_2_). The 5′-phosphatase targets primarily phosphatidylinositol (4,5)-bisphosphate (PI(4,5)P_2_). Synj1-mediated phosphoinositide remodeling has been shown to facilitate synaptic vesicle recycling and autophagosome maturation^27–29^. Despite the reported role of phosphoinositide in endosomal sorting^29–31^, it remains unclear whether and how Synj1 is involved in presynaptic endosomal function. Earlier studies have reported swelling of early endosomes in the soma of cortical neurons overexpressing Synj1^32^, and endosomal abnormalities have also been observed in Synj1-deficient cells^33,34^. Considering that Synj1 is broadly expressed, it remains unclear whether these defects are responsible for the selective vulnerability of the dopaminergic synapses.

Dopaminergic synapses are among the least well understood despite their clinical relevance to PD. Studies in animal models of PD have shown that dysfunctional dopamine release precedes neurodegeneration^1,2,35–39^ but the mechanisms that maintain synaptic dopamine release is not fully understood. Intriguingly, deletion of Synj1 substantially slows synaptic vesicle endocytosis, yet little effect was observed in basal glutamatergic or GABAergic transmission^40,41^. How Synj1 regulates dopamine release remains unclear. Unlike glutamatergic synapses, which rely on adjacent glial cells for neurotransmitter recycling^42^, dopaminergic synapses depend on the axonal dopamine transporter (DAT). Deleting DAT results in 75% reduction in the evoked dopamine release, suggesting its essential role in dopamine storage^43^. The dopamine D2 receptor short isoform (D2S) is a G_αi/o_-coupled class-A GPCR that acts as an autoreceptor to gate dopamine release^44,45^ by inhibiting voltage-gated Ca^2+^ channels^46–48^ and dopamine synthesis^49–51^. The D2S directly interacts with DAT and is expressed exclusively in dopaminergic axons^52,53^. Disrupting this interaction results in a lack of DAT function in mice^54^, placing D2S as a critical upstream regulator for synaptic dopamine release. As plasma membrane cargos, both DAT and D2S are subject to endocytic trafficking. While we know little about the D2S, the VPS35 retromer as well as several protein kinases and GTPases have been shown to maintain DAT endosomal sorting and surface trafficking^39,55–60^. Patients with early-stage PD often exhibit altered levels of DAT and D2 receptors^61–63^, but we still lack an understanding in how their altered expression or trafficking contribute to motor deficits in PD.

Here, we identified a novel signaling pathway that regulates presynaptic endosomal sorting of DAT and D2S to maintain synaptic dopamine release. We show that Synj1 remodels endosomal PI4P to facilitate the recruitment of the VPS35 retromer to presynaptic endosomes (Supplemental Fig. S1). Mice with complete deletion of Synj1 in dopamine neurons exhibit endosomal swelling, cargo retention, reduced dopamine release and locomotor impairments. We further show reduced VPS35 expression in Synj1-depleted axons and dopamine-induced co-clustering of Synj1 and VPS35, supporting their broad impact on surface cargo trafficking. These findings reveal novel endosome-associated quality control mechanisms at the dopamine synapse contributing to dopaminergic vulnerability in early PD pathogenesis.

## Results

### Synj1 controls presynaptic D2S and DAT surface availability in dopamine neurons.

**Figure 1:**
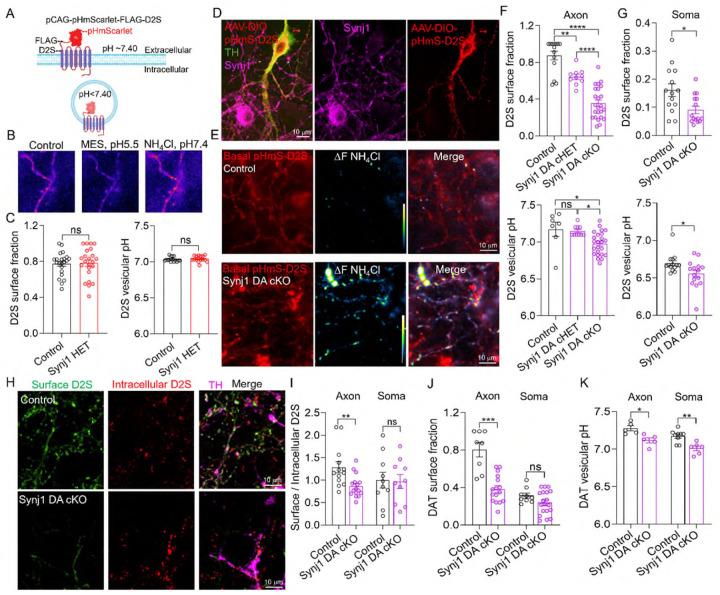
Synj1 controls presynaptic D2S and DAT surface levels in dopamine neurons. **A**) Schematic of the pHmScarlet-D2S construct and its working mechanism. pHmScarlet is a red-shifted, pH-sensitive fluorophore that fluoresces at neutral pH and is quenched in acidic compartments. **B)** Representative images of axon expressing pHmScarlet-D2S during perfusion with MES buffer (pH 5.5) and NH_4_Cl (pH 7.4). **C**) Quantification of basal D2S surface fraction and vesicular pH in littermate control (*Synj1*+/+, n=22) and *Synj1* HET (*Synj1*+/−, n=23) neurons using MES-NH_4_Cl perfusion, with vesicular pH calculated based on the Henderson-Hasselbalch equation. Data are from four batches of independent cultures (Student’s *t*-test). **D)** Immunofluorescence images of AAV9-SYNp-DIO-pHmScarlet-FLAG-D2S expressed in cultured dopaminergic neurons from Synj1 DA cKO mice. Scale bar, 10 μm. **E)** Representative images of baseline pHmScarlet-D2S fluorescence and the ΔF response to NH_4_Cl in control (*DAT-Cre*) and *Synj1* DA cKO axons expressing AAV-SYNp-DIO-pHmScarlet-D2S. NH_4_Cl ΔF shows intracellular D2S levels. Scale bar, 10 μm. **F-G)** Quantification of D2S surface fraction and vesicular pH in axons (**F**) and soma (**G**) of Control (*DAT-Cre*) (n=17 axons, n=13 soma) and *Synj1* DA cKO neurons (n=24 axons, n=14 soma) (Mann-Whitney tests). **H**) Representative immunofluorescence images of surface (non-permeabilizated) and intracellular (permeabilized) FLAG-D2S in littermate control (*Synj1*^flox/flox^) and *Synj1* DA cKO neurons. Scale bar, 10 μm. **I)** Quantification of surface-to-intracellular D2S ratio in axons and soma (control, *Synj1*^flox/flox^: n=13 axon, n=10 soma; *Synj1* DA cKO: n=6 axon, n=10 soma). Data from two independent culture preparations (Student’s *t*-test). **J, K)** Quantification of DAT surface fraction and vesicular pH in axons (J) and soma (K) of control (*DAT-Cre*, n=17 axons, n=13 soma) and *Synj1* DA cKO neurons (n=24 axons, n=14 soma) (Mann-Whitney tests). Data are shown as the Mean ± SEM with individual data points. ns, not significant; *p < 0.05, **p < 0.01, ****p < 0.0001

In previous work, *Synj1* heterozygous mice showed altered dopamine metabolism and impaired DAT trafficking^64^. As the D2S autoreceptor is a key regulator of dopamine release and may act upstream of these processes^65^, we examined whether and how Synj1 regulates D2S. Because of the lack of reliable approaches to label endogenous axonal D2S, we generated a pH-sensitive reporter by fusing the red-shifted pHmScarlet (pHmS) to the extracellular N-terminus of FLAG-tagged D2S (Fig. 1A). This probe, when expressed in ventral midbrain neurons, fluoresces at neutral pH on the cell surface and is quenched in acidic compartments, allowing quantification of the surface fraction of D2S and the intracellular pH of D2S-containing vesicles (Fig. 1A).

To validate and characterize this new reporter, we first determined the pKa of pHmS-D2S by perfusing buffered Tyrodes’ solutions across a range of pH values. The best fitted pKa of pHmS-D2S, determined using the Henderson-Hasselbalch equation, was 7.50 (Supplemental Fig. S2A), consistent with the reported values for the untagged pHmScarlet^66^ and ideal for analyzing surface proteins. We next tested whether the pHmS-tagged D2S receptor retains normal function. D2S activation inhibits presynaptic Ca^2+^ entry through Gα_i/o_-coupled suppression of voltage-gated Ca^2+^ channels. We therefore co-expressed GCaMP6f with either pHmS-D2S or FLAG-D2S and measured Ca^2+^ responses to electrical stimulation before and after 10 μM dopamine perfusion (Supplemental Fig. S2B–D). Dopamine induced comparable reductions in Ca^2+^ signals in both conditions (Supplemental Fig. S2C–D), indicating that the tagged receptor is functionally intact.

Following validation, we quantified basal D2S surface trafficking by expressing the pHmS-D2S in cultured ventral midbrain neurons from *Synj1* HET and littermate control (*Synj1+/+*) mice. Surface and intracellular receptor pools were measured by sequential perfusion with MES (pH 5.50) and NH_4_Cl (pH 7.40) solutions. At baseline, *Synj1* HET axons showed no difference in pHmS-D2S surface fraction or vesicular pH compared to controls (Fig. 1C).

Our *post hoc i*mmunofluorescence analysis showed that most of the midbrain neurons expressing pHmS-D2S driven by the CAG promoter were non-dopaminergic as indicated by the absence of the dopaminergic marker, tyrosine hydroxylase (TH). To specifically determine D2S regulation in dopamine neurons, we generated a condition-ready mouse line (*Synj1*^*flox/flox*^) with *loxP* sites inserted to both ends of the critical exon 4. When crossed to the *DAT*^*IRES-Cre*^ (*DAT-Cre*) driver line, offspring (*Synj1* DA cKO mice) exhibit complete deletion of *Synj1* in all dopamine neurons (Supplemental Fig. S3A, B), which was verified in the ventral midbrain culture (Fig. 1D). The *Synj1* DA cHet (*Synj1*^*flox/-*^*; DAT-Cre*^*+/−*^) or *Synj1* DA cKO mice (*Synj1*^*flox/flox*^*; DAT-Cre*^*+/−*^) cultures were transduced with an AAV9-SYNp-DIO-pHmS-D2S to ensure *Cre*-dependent expression in dopamine neurons. We found that compared to control (*DAT-Cre*) neurons that exhibited about 90% surface pHmS-D2S in the axons, the *Synj1* DA cHet axons showed a 20–30% reduction and the *Synj1* DA cKO axons showed a 60% reduction (Fig. 1E–F), with majority of the pHmS-D2S accumulated intracellularly (Fig. 1E–F). Intracellular pHmS-D2S vesicles in these neurons also exhibited lower pH (Fig. 1F), consistent with impaired trafficking. At soma, pHmS-D2S showed ~15% surface expression in control neurons, and the intracellular vesicles were more acidic than those in axons (Fig. 1G). In *Synj1* DA cKO neurons, the surface fraction and vesicular pH of pHmS-D2S were modest but significantly reduced (Fig. 1G). We independently validated these findings using surface and intracellular immunostaining of FLAG-D2S, although differences in the soma were not detected (Fig. 1I).

In our previous study, we examined DAT-pHluorin - a similar pH sensitive sensor for DAT, and found a ~15% reduction of its surface fraction in *Synj1* HET axons^64^. We now analyzed *Synj1* DA cKO neurons using AAV9-SYNp-DIO-DAT-pHluorin. Consistent with D2S, the DAT-pHluorin surface fraction was reduced to ~30% in *Synj1* DA cKO axons at baseline, accompanied by a decrease in vesicular pH (Fig. 1 J, K). At soma, the DAT-pHluorin vesicular pH was reduced but the surface fraction was only trending to be reduced (Fig. 1J, K).

Collectively, these results showed that Synj1 dose-dependently regulates presynaptic surface availability of the exogenously expressed D2S and DAT in dopaminergic axons.

### Dopamine-induced D2S trafficking requires Synj1.

**Figure 2:**
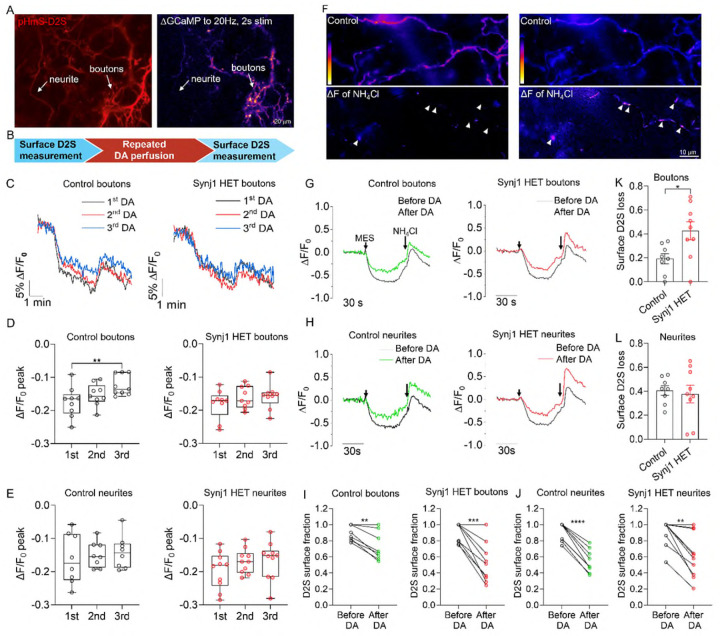
Dopamine-induced trafficking of D2S to the presynaptic surface requires Synj1. **A)** Representative images of ventral midbrain neuronal processes expressing pHmScarlet-D2S and GCaMP6f. ΔF GCaMP6f responses to a 20 Hz, 2 s stimulation were used to identify presynaptic boutons and distinguish them from neurites (white arrows). Scale bar, 10 μm. **B**) Schematic of the live imaging workflow. D2S surface fraction was measured in the same neuron before and after three repeated perfusions of 10 μM dopamine (DA) using the MES-NH_4_Cl perfusion. **C**) Representative traces of pHmScarlet-D2S response at boutons during three repeated DA perfusions (color-coded) in littermate control (*Synj1*^+/+^) and *Synj1* HET (*Synj1*^+/−^) neurons. **D, E**) Quantification of peak ΔF/F_0_ during DA perfusion in boutons (D) and neurites (E) of control and *Synj1* HET neurons. Paired comparisons between the 1^st^ and 3^rd^ DA perfusion are shown for each condition. **F**) Representative images from MES-NH_4_Cl perfusion before and after DA treatment. Top panels show baseline pHmScarlet-D2S fluorescence; bottom panels show ΔF following NH_4_Cl, revealing intracellular D2S (arrowheads). Scale bar, 10 μm. **G, H**) Representative traces of D2S surface fraction measurements in boutons (**G**) and neurites (**H**) of control and *Synj1* HET neurons. (**I, J**) Quantification of D2S surface fraction before and after repeated DA perfusion in boutons (I) and neurites (J) of control and *Synj1* HET neurons (control bouton: n=8, neurite: n=8; *Synj1* HET bouton: n=9, neurite: n=10). Paired comparisons are shown for each condition. (**K-L**) Comparison of DA-induced changes in D2S surface fraction between control and *Synj1* HET neurons in boutons (K) and neurites (L). Data are presented as mean ± SEM with individual data points shown. Paired two-tailed student’s *t*-tests were used for within-neuron comparisons, and unpaired two-tailed Student *t*-tests were used for between-group comparisons. *p < 0.05, **p < 0.01, ***p < 0.001, ****p < 0.0001

Reduction in basal pHmS-D2S surface levels in dopamine neurons, but not in non-dopaminergic *Synj1* HET neurons, suggest a role for dopamine in D2S surface trafficking. We next sought to determine how dopamine regulates axonal D2S and whether dopamine-induced pHmS-D2S trafficking depends on Synj1. To focus on presynaptic release sites, we co-expressed GCaMP6f and pHmS-D2S in *Synj1* HET and littermate control neurons and mapped the activity-dependent (20 Hz, 2 sec stimulation) GCaMP6f hotspots as active boutons (Fig. 2A). The pHmS-D2S surface fraction was then measured at these boutons and non-bouton areas (denoted as neurite) before and after repeated dopamine perfusion (Fig. 2B). Dopamine perfusion induced a rapid reduction in pHmS-D2S fluorescence at presynaptic boutons (Fig. 1C), consistent with a loss of surface receptors.

In control boutons, the magnitude of dopamine-induced fluorescence loss progressively decreased across repeated perfusion, indicating a reduction in pHmS-D2S internalization with repeated exposure (Fig. 2C, D). In contrast, this attenuation was not observed in *Synj1* HET neurons, where boutons exhibited a sustained loss of surface receptors (Fig. 2C, D). Consistent with this, dopamine perfusion resulted in a greater reduction in pHmS-D2S surface fraction in *Synj1* HET boutons compared to controls (Fig. 2G, I, K), whereas no difference was observed in the neurites, indicating that the trafficking defect is specific to presynaptic release sites (Fig. 2H, J, L).

Together, these results suggest that Synj1 is required for ligand-induced trafficking and surface maintenance of pHmS-D2S at presynaptic release sites.

### Synj1 regulates the D2S-dependent dopamine release and signaling.

**Figure 3:**
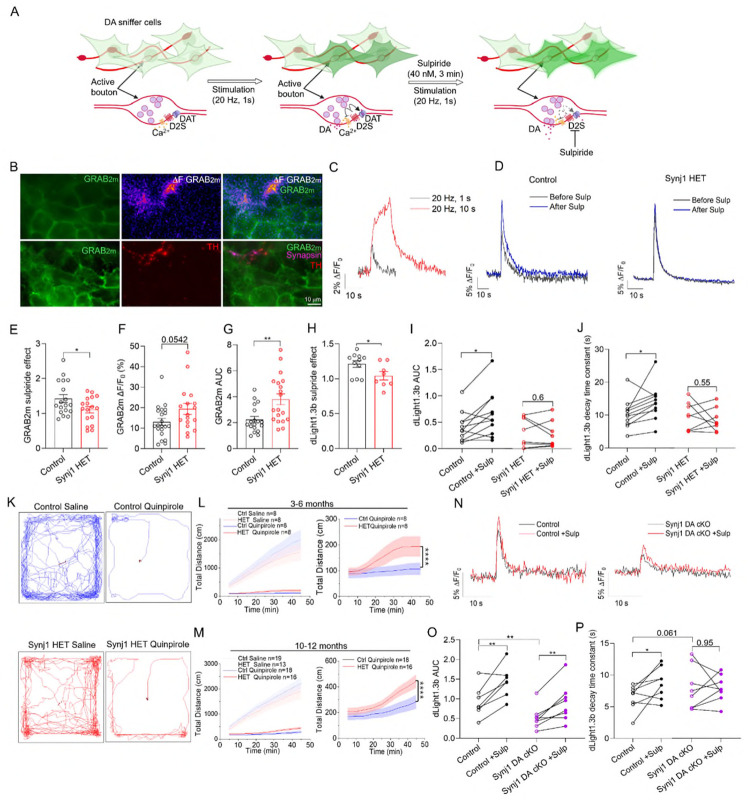
Synj1 loss impairs D2 autoreceptor control of dopamine release in vitro and in vivo. **A**) Schematic of the experimental setup for measuring presynaptic dopamine release in ventral midbrain neuron-sniffer cell co-cultures. Action potential-evoked, Ca^2+^-dependent dopamine release at presynaptic boutons under basal D2S autoreceptor regulation and is modulated with dopamine transporter (DAT)-mediated reuptake. Released dopamine is detected by overlaid sniffer cells expressing GRAB_DA2m_ or dLight1.3b. **B**) Representative images of electrically evoked dopamine release at presynaptic boutons. Top: GRAB_DA2m_ baseline fluorescence, ΔF response to 20 Hz, 1 s stimulation, and overlay. Bottom: *post-hoc* immunofluorescence showing sniffer cells (GFP), dopaminergic axons (TH), and synapsin1/2 marking presynaptic boutons. **C**) Representative GRAB_DA2m_ traces from the same release sites in response to 20 Hz, 1 s (black) and 20 Hz, 10 s (red) stimulation. **D**) Representative GRAB_DA2m_ traces from littermate control (*Synj1*+/+) and *Synj1* HET (*Synj1*+/−) neurons in response to 20 Hz, 1 s stimulation before (black) and after (blue) sulpiride (40 nM, 2 min perfusion). **E-G**) Quantification of sulpiride-induced changes (fold change) in GRAB_DA2m_ peak release (E), ΔF/F_0_ (F), and area under the curve (AUC) (G) in control (n=18 release sites) and *Synj1* HET (n=17 release sites) neurons. Data from five independent co-culture preparations. **H-J**) Quantification of sulpiride-induced changes (fold change) in dLight1.3b peak release (H), AUC (I), and decay time constant (J). Data from two independent co-culture preparations. **K-M)** Locomotor response to the D2-like agonist quinpirole. Control and *Synj1* HET male littermate (3–6 months) received intraperitoneal injections of saline or quinpirole (0.5 mg/kg), and locomotor activity (total distance traveled) was recorded over 45 min in an open field. Cumulative distance is shown; quinpirole-treated groups are plotted on a separate scale. (M) Same analysis in an independent cohort of older mice (10–12 months). **N-P)** dLight1.3b measurements of dopamine release in control (*DAT-Cre*) and *Synj1* DA cKO neurons. Representative traces in response to 20 Hz, 1 s stimulation before (black) and after (blue) sulpiride (40 nM, 2 min perfusion). **O-P)** Quantification of sulpiride-induced changes (fold change) in AUC (O) and decay time constant (P). Data are presented as mean ± SEM with individual data points shown. Statistical significance determined using paired or unpaired two-tailed Student’s *t*-tests, as appropriate. For panel L-M, two-way repeated-measures ANOVA was used. ns, not significant; *p < 0.05, **p < 0.01

To investigate the impairment of endogenous D2S in Synj1-deficient neurons, we monitored evoked dopamine release using sniffer cells expressing GRAB_DA2m_ or dLight1.3b sensors as previously described^67–69^ (Fig. 3A). Field electrical stimulation produced robust and scalable dopamine transients from boutons (Fig. 3B–C). The endogenous D2S autoreceptor function was assessed by comparing the evoked dopamine release before and after the application of a D2-like antagonist sulpiride (40 nM). In control neurons, sulpiride increased release amplitude in GRAB_DA2m_ cells by *~*40%, consistent with release of the D2S-mediated inhibition (Fig. 3D–G). In contrast, this response was reduced to 10% in *Synj1* HET neurons, indicating an impaired D2S autoreceptor function (Fig. 3D–G). This deficit was verified using dLight1.3b-based measurements (Fig. 3H–I). To assess the D2S-dependent regulation of DAT function in dopamine clearance, we examined the decay time constant of dopamine transients. In control neurons, sulpiride increased the decay time constant, consistent with an inhibition of DAT (Fig. 3J). This effect was absent in *Synj1* HET neurons (Fig. 3J), indicating a loss of D2S-DAT cooperation. The significantly higher baseline GRAB_DA2m_ fluorescence and a trend of faster decay in the evoked responses in *Synj1* HET neurons (Supplemental Fig. S4) were in line with our previous findings using dLight1.3b-based measurements, reflecting an altered DAT function^64^.

We next tested whether the D2S deficit is observed *in vivo*. Two cohorts of *Synj1* HET and littermate control mice at 3–6 months and 10–12 months were intraperitoneally injected with either saline or a D2-like agonist, quinpirole (0.5 mg/kg), and locomotor activity was monitored over 45 min. Quinpirole suppressed locomotion in all groups, but this effect was significantly reduced in male *Synj1* HET mice compared to littermate controls at both ages tested (Fig. 3K–M). In contrast, female *Synj1* HET mice showed responses comparable to controls (Supplemental Fig. S5), indicating a sex-dependent effect.

To determine how complete loss of Synj1 affects D2S-dependent dopamine release, we compared evoked responses between *Synj1* DA cKO neurons and *DAT-Cre* controls. As expected, sulpiride increased dopamine release and prolonged the decay of dopamine transients in control neurons (Fig. 3N–P). In *Synj1* DA cKO neurons, the effect of sulpiride on dopamine release was preserved, however, its effect on decay was absent, indicating a lack of cooperation between D2S and DAT (Fig. 3N–P). Notably, evoked dopamine transient decreased substantially in *Synj1* DA cKO neurons, suggesting a severe deficit at the synapse.

Together, these results show that Synj1 is required for D2 autoreceptor control of dopamine release and clearance, linking presynaptic trafficking defects to altered dopaminergic signaling *in vitro* and *in vivo*.

### Synj1 deletion in dopamine neurons disrupts motor behavior, striatal dopamine storage and DAT expression.

**Figure 4:**
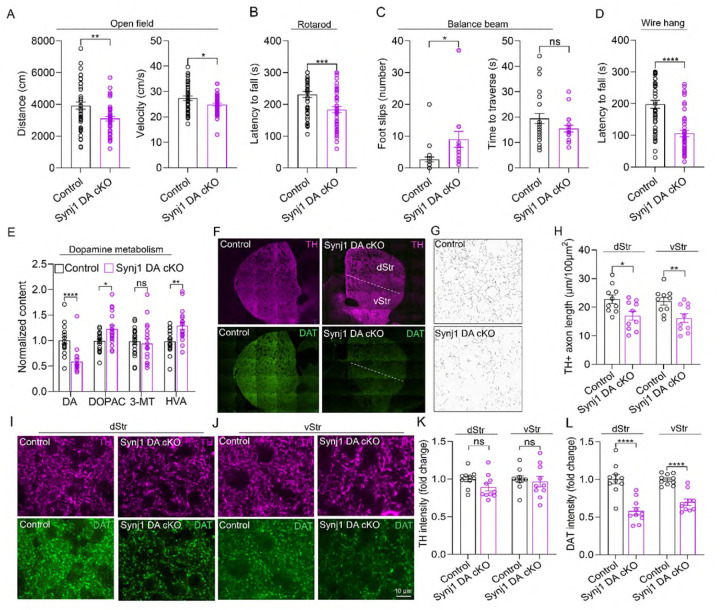
*Synj1* DA cKO mice exhibit motor impairment, loss of striatal dopamine and reduced DAT expression. *Synj1* DA cKO and control (*Synj1*^flox/flox^) littermate mice (male and female, 3 months) were subjected to behavioral and biochemical analyses. **A)** Total distance travelled and velocity of episodic movement in a 30-minute open field test. **B)** Time to fall on the accelerated Rotarod. **C)** Number of foot slips and time to traverse the beam on the balance beam test. **D)** Latency to fall on a 5-minute wire hang test. **E)** Normalized content of dorsal striatal dopamine and metabolites including DOPAC (3,4-dihydrophenylacetic acid), HVA (homovanillic acid), and 3-MT (3-methoxytyramine). **F)** Representative 20× images of dorsal and ventral striatum from control and *Synj1* DA cKO mice showing TH and DAT immunostaining (white dotted line separates dorsal and ventral regions). **G)** Axonal skeletonization images generated from TH staining using Fiji to visualize axonal coverage. **H)** Quantification of TH-positive axonal length density (μm/100 μm^2^) in dorsal and ventral striatum**. I-J)** Representative images of TH and DAT immunostaining in dorsal (I) and ventral (J) striatum from control and *Synj1* DA cKO mice. **K-L)** Quantification of images of TH fluorescence intensity (K) and DAT fluorescence intensity (L) in dorsal and ventral striatum. Data are shown as mean ± SEM. Statistical significance was determined by two-tailed unpaired Student’s *t*-test with Welch’s correction, unless otherwise indicated. ns, not significant; *p < 0.05, **p < 0.01, ***p < 0.001, ****p < 0.0001. Each symbol represents one mouse or one brain.

To determine whether Synj1 deletion in dopamine neurons has any behavioral consequences, we conducted a battery of locomotor tests for *Synj1* DA cKO mice and their littermate (*Synj1* ^flox/flox^ mice) containing both sexes. Complete deletion of Synj1 leads to perinatal lethality in mice^40^. However, *Synj1* DA cKO mice exhibited normal lifespan, fertility and weight gain (Supplemental Fig. S3B). At 3 months, we found that the *Synj1* DA cKO mice showed reduced locomotor activity in the open field, with decreased total distance and episodic movement velocity (Fig. 4A). These mice also showed impaired motor coordination with reduced latency to fall on the accelerating rotarod compared to controls (Fig. 4B). In the balance beam test, the *Synj1* DA cKO mice showed increased foot slips without a change in traversal time (Fig. 4C). Four-limb muscle strength was examined in a wire hang test, in which *Synj1* DA cKO mice showed reduced latency to fall, indicating decreased muscle strength (or hypotonia) (Fig. 4D). Similar locomotor deficits were observed in a younger (1–2 months) cohort; however, no differences were detected in wire hang and balance beam measurements (foot slips and traversal time) (Supplemental Fig. S6), suggesting progressive decline in motor function. Sex difference was not observed in any of the above measures, except that hydrocephaly was observed incidentally in the female *Synj1* DA cKO mice and these mice were excluded from the behavioral study. A separate analysis was conducted for littermate wildtype (WT) and *DAT-Cre*^*+/−*^ mice, and no significant differences were observed in all behavioral measurements (Supplemental Fig. S7), indicating that the *DAT-Cre* transgene does not contribute to the observed phenotypes.

We next examined whether tissue dopamine content and metabolism were altered in *Synj1* DA cKO mice. Freshly isolated dorsal striata from a subset of the behavioral mice were subject to high-performance liquid chromatography (HPLC) analysis. The *Synj1* DA cKO mice showed a nearly 50% reduction in dopamine, a 25% increase in DOPAC and HVA, while the 3-MT remained unchanged, indicating decreased dopamine storage and increased intracellular dopamine turnover (Fig. 4E). Sex difference was not noted. To determine whether these changes reflect dopaminergic axon degeneration, we quantified TH-positive projections. TH-positive axon length per area was reduced by 25% in both dorsal and ventral striatum (Fig. 4F–H), suggesting functional defects other than axonal degeneration contribute to the loss of tissue dopamine content in the dorsal striatum. Because dopamine storage critically depends on DAT, we next examined DAT expression levels in the striatum. DAT immunofluorescence intensity was reduced by 40% in the dorsal striatum and 35% in the ventral striatum of the *Synj1* DA cKO mice (Fig. 4I–J, K). This difference was not observed for the *DAT-Cre* mice when compared to littermate controls (Fig. S6). Interestingly, the TH immunofluorescence intensity was unchanged (Fig. 4I–K), indicating higher TH expression per axonal fiber. Another interesting pathological finding is the emergence of TH-positive cell bodies in the striatum of *Synj1* DA cKO mice, which was more numerous in the dorsal versus ventral striatum (Supplemental Fig. S8). The functional role of these TH-positive striatal neurons remains unclear at present, although similar cells have been reported in a few other PD models^24,70–72^.

Together, these data show that Synj1 deletion in mice impairs motor coordination, dopamine storage, metabolism, as well as DAT expression *in vivo*.

### Synj1 deletion in dopamine neurons induces dystrophic DAT clusters associated with enlarged EEA1-positive endosomes in the striatum

**Figure 5:**
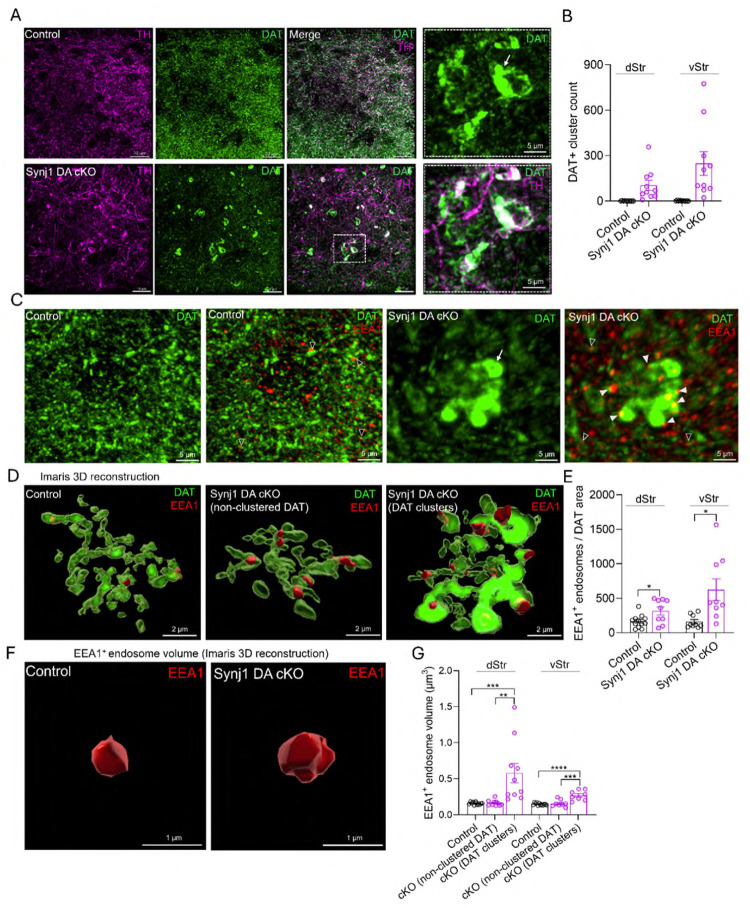
Increased DAT-associated endosomal density and volume in the *Synj1* DA cKO brains. **A)** Representative images showing DAT distribution in control and *Synj1* DA cKO striatum, with accumulation into large, discrete axonal clusters (white arrow). Scale bar, 20 μm. Higher-magnification view of the boxed region. Scale bar, 5 μm. **B)** Quantification of DAT-positive cluster number in dorsal and ventral hemi striatum. Each symbol is a brain. **C)** Representative images of DAT and EEA1 immunostaining in striatal sections from littermate control (*Synj1*^flox/flox^) and *Synj1* DA cKO mice. Arrow indicates DAT clusters. Filled arrowheads denote EEA1+ endosomes associated with DAT clusters; open arrowheads denote EEA1+ endosomes associated with non-clustered DAT. Scale bar, 5 μm. **D)** Representative Imaris 3D reconstructions showing EEA1+ endosome association with DAT in control, *Synj1* DA cKO (non-clustered DAT), and *Synj1* DA cKO (DAT clusters). **E)** EEA1+ endosomes count per DAT area (μm^2^) in dorsal and ventral striatum of control and *Synj1* DA cKO mice. **F)** Representative Imaris 3D reconstructions of EEA1+ endosomal volume in control and *Synj1* DA cKO striatum. **G)** Quantification of EEA1+ endosome volume (μm^3^) in control, *Synj1* DA cKO (non-clustered DAT), and *Synj1* DA cKO (DAT clusters) in dorsal and ventral striatum. Data are shown as mean ± SEM with individual data point indicating an image. Statistical significance was determined using unpaired two-tailed Student *t*-tests or one-way ANOVA with Tukey’s multiple comparisons test. *p < 0.05, **p < 0.01, ***p < 0.001, ****p < 0.0001

In addition to reduced DAT expression, we observed large DAT-positive clusters co-labelled with TH in the striatum of the *Synj1* DA cKO mice (Fig. 5A, B). Similar DAT clusters were reported in the dorsal striatum of the PARK20 (*Synj1* R258Q knockin) mice, suggesting axonal dystrophy. To further determine the subcellular localization of these DAT clusters, we performed immunofluorescent labeling for the early endosome marker, EEA1. Using Imaris-based 3D reconstruction, we defined DAT surface and clusters and quantified their association with EEA1-positive endosomes. Although not all DAT structures were associated with EEA1, the number of EEA1-positive endosomes associated with DAT was significantly increased in the striatum of *Synj1* DA cKO mice (Fig. 5C, D). This *in vivo* observation is in line with the *in vitro* data showing increased intracellular DAT in Synj1 deficient dopaminergic axons (Fig. 1J). We then quantified the volume of EEA1-positive endosomes association with DAT and found that those associated with dystrophic DAT clusters in *Synj1* DA cKO mice were significantly larger (Fig. 5E–G). Additionally, while the amount of DAT clusters and DAT-associated endosomes were higher in the ventral striatum, the volume of DAT cluster-associated endosomes was larger in the dorsal striatum, suggesting region-specific regulation.

### Synj1 is required for PI4P-dependent recruitment of VPS35 and Rab7a to D2S-containing endosomes.

**Figure 6:**
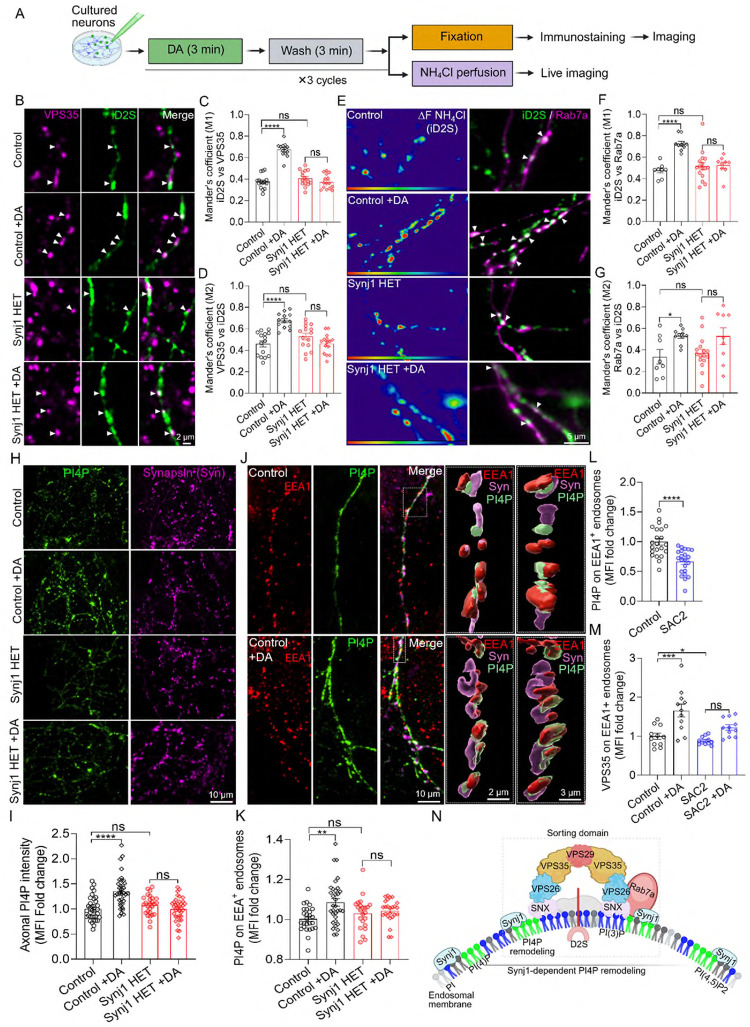
Dopamine-induced recruitment of VPS35 and Rab7a to D2S-containing endosomes and increases in PI4P requires Synj1. **A)** Schematic of the repeated dopamine (DA) perfusion and imaging workflow. **B-D)** Ventral midbrain control (*Synj1*^*+/+*^) and Synj1 HET (*Synj1*^*+/−*^) neurons expressing FLAG-D2S were perfused with vehicle or 10 μM dopamine (DA), followed by surface FLAG blocking and immunolabeling for VPS35 and FLAG. Representative confocal images of axons show colocalization between intracellular FLAG-D2S (iD2S, green) and VPS35 (magenta) across conditions (B). Scale bar, 2 μm. Colocalization is quantified as Mander’s coefficient M1 (C; iD2S with VPS35) and M2 (D; VPS35 with iD2S). **E-G)** Control and Synj1 HET neurons co-expressing pHluorin-D2S and tdTomato-Rab7a were perfused with vehicle or 10 μM DA, followed by live NH_4_Cl perfusion to reveal intracellular D2S. Representative live axonal images show ΔF signals corresponding to intracellular pHluorin-D2S (iD2S) and Rab7a (E). Scale bar, 5 μm. Colocalization between iD2S and Rab7a is quantified as M1 (F) and M2 (G). Each data point represents analysis of axons within one field of view. Data are from two independent culture preparations. **H, I)** Representative confocal images of axonal PI4P (green) and Synapsin (magenta) (H) and quantification of axonal PI4P intensity (I) in control and Synj1 HET neurons following vehicle or 10 μM DA perfusion. Scale bar, 10 μm. **J-K)** Representative images of axonal EEA1-positive endosome associated with PI4P (J). Scale bar, 10 μm. A boxed axonal region is rendered in 3D using Imaris to visualize Synapsin-defined presynaptic structures, EEA1-positive endosomes, and associated PI4P signal (top and side views). Scale bar, 2 μm or 3 μm. Quantification of axonal PI4P intensity on EEA1+ endosomes is shown in (K). **L)** Quantification of PI4P levels on EEA1-positive endosomes in N2A cells expressing GFP or SAC2-GFP. **M)** Quantification of VPS35 enrichment on EEA1-positive endosomes in N2A cells co-expressing FLAG-D2S with GFP (control) or SAC2-GFP under basal conditions or following DA perfusion. **N)** Model illustrating Synj1-dependent control of endosomal PI4P required for recruitment of VPS35 and Rab7a to D2S-containing endosomes. Data are presented as mean ± SEM with individual data points shown. Statistical significance was determined using unpaired two-tailed Student’s *t*-tests or two-way ANOVA with Tukey’s multiple comparisons test. ns, not significant; *p < 0.05, ***p < 0.001, ****p < 0.0001

Our data so far suggest that DAT and D2S are likely trapped in presynaptic endosomes when Synj1 is deficient. We next sought to investigate the Synj1 downstream signaling responsible for the observed trafficking defects. Endosomal sorting is typically mediated by the retromer complex. We therefore asked whether Synj1 regulates endosomal sorting of D2S through a VPS35-dependent pathway. In WT midbrain neurons expressing FLAG-D2S, intracellular D2S showed ~40% colocalization with endogenous VPS35 at baseline (Fig. 6A–C). Repeated dopamine (10 μM) perfusion increased this colocalization, as measured by both Mander’s coefficient (M1 and M2) and Pearson’s correlation (Supplemental Fig. S9). In contrast, this dopamine-induced increase was absent in *Synj1* HET neurons, indicating that Synj1 is required for dopamine-dependent recruitment of VPS35 to FLAG-D2S-containing endosomes.

Because VPS35 recruitment is associated with endosomal maturation, we next examined Rab7a-positive endosomes, which supports retromer-mediated sorting^73–75^. In neurons co-expressing pHluorin-D2S and TdTomato-Rab7a, we quantified Rab7a colocalization with intracellular D2S in live neurons following NH_4_Cl perfusion. Dopamine increased D2S-Rab7a colocalization in WT axons (M1 and M2), whereas this response was not observed in *Synj1* HET axons (Fig. 6E–H, Supplemental Fig. S9), indicating impaired recruitment of VPS35-Rab7a to D2S-containing endosomes.

Endosomal lipids play a key role in the recruitment of molecular machinery during sorting. The predominant phosphoinositide lipid species on the endosomal membrane are phosphatidylinositol 3-phosphate (PI3P). Recent studies suggested the formation of a PI3P-to-PI4P gradient on early endosomes during cargo sorting and recycling^76,77^. Low levels of PI(4,5)P_2_ have also been reported to present on endosomes that play a role in cargo sorting^78^. To determine the phosphoinositide dynamics essential for VPS35 and Rab7a recruitment we measured axonal PI4P levels using antibody labeling in cultured ventral midbrain neurons. We found that dopamine perfusion increased PI4P within Synapsin-defined axonal regions in WT neurons, whereas this increase was absent in *Synj1* HET neurons (Fig. 6H, I). Failure to recruit Rab7a suggests defects in endosomal maturation. We then focused on EEA1-positive early endosomes, where D2S accumulates prior to sorting. Measurement of PI4P fluorescence intensity within EEA1-positive endosomes showed a similar dopamine-dependent increase in WT control but not in *Synj1* HET neurons (Fig. 6J, K).

To directly test whether PI4P is required for VPS35 recruitment, we used N2A cells expressing D2S-FLAG together with GFP or SAC2-GFP, an endosomal PI4P phosphatase. Immunostaining confirmed that SAC2 expression reduced PI4P levels within EEA1-positive endosomes (Fig. 6L). Dopamine perfusion increased VPS35 enrichment on EEA1-positive endosomes in control cells, whereas this increase was attenuated in SAC2-expressing cells (Fig. 6M), indicating that PI4P is needed for VPS35 recruitment to D2S-containing endosomes. Dopamine perfusion increased EEA1-positive endosome area and reduced endosome number per cell, whereas these measures were unchanged in SAC2-expressing cells (Supplemental Fig. S10).

Together, these data support a model (Fig. 6N) in which Synj1-mediated endosomal PI4P remodeling is required for dopamine-induced recruitment of VPS35 and Rab7a to D2S-containing endosomes.

### VPS35 rescues presynaptic trafficking defects in Synj1-deficient neurons.

**Figure 7:**
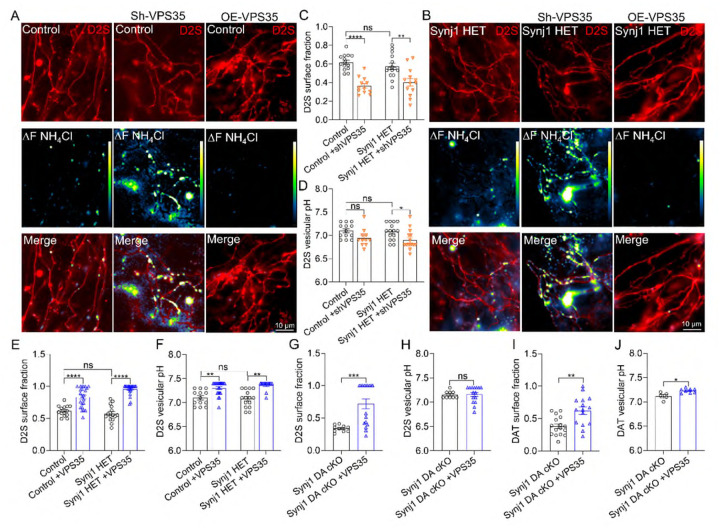
VPS35 regulates presynaptic D2S and DAT surface availability and rescues Synj1-dependent defects. **A, B)** Representative confocal images of live neuronal axons expressing pHmS-D2S, showing baseline fluorescence, intracellular D2S revealed by NH_4_Cl, and merged images. Scale bar, 10 μm. **C-F)** Quantification of axonal D2S surface fraction and vesicular pH in control (*Synj1*^*+/+*^, n=14), Synj1 HET (*Synj1*^*+/−*^, n=15) midbrain neurons expressing pHmScarlet-D2S alone, with VPS35 knockdown (BFP-VPS35 shRNA) (C, D), or with VPS35 overexpression (VPS35-eGFP) (E, F). Data are from three independent culture preparations. **G-J)** Quantification of axonal D2S surface fraction (G) and vesicular pH (H), DAT surface fraction (I), and vesicular pH (J) in *Synj1* DA cKO neurons (n=10) with or without VPS35 overexpression (AAV2/5-DIO-VPS35; n=17). Data are shown as mean ± SEM with individual data points. Statistical significance was determined using two-way ANOVA with Tukey’s multiple comparisons test (G-F) or unpaired two-tailed Student’s *t*-tests (G-J). ns, not significant; *p < 0.05, **p<0.01, ***p < 0.001, ****p < 0.0001

To determine whether the impaired VPS35 recruitment is the rate-limiting step downstream of Synj1-mediated presynaptic sorting, we examined the basal axonal pHmS-D2S surface fraction under various combinations of genetic backgrounds. First, we modulated VPS35 levels in cultured WT midbrain neurons using shRNA-mediated knockdown or VPS35 overexpression (Supplemental Fig. S11). VPS35 knockdown reduced pHmS-D2S surface fraction in *Synj1* HET axons to 35% (Fig. 7A–C), comparable to what was found in *Synj1* DA cKO neurons (Fig. 1F). Conversely, VPS35 overexpression increased pHmS-D2S surface fraction in *Synj1* HET neurons, accompanied by an increase in vesicular pH (Fig. 7E, F). We next manipulated VPS35 levels in *Synj1* HET neurons. While overexpressing VPS35 enhanced pHmS-D2S surface expression, VPS35 knockdown in *Synj1* HET neurons did not further reduce surface pHmS-D2S (Fig. 7B), indicating that Synj1 and VPS35 work in the same pathway.

To further test the Synj1-VPS35 signaling axis, we asked whether increasing VPS35 can compensate for Synj1 loss and rescue presynaptic trafficking deficits in *Synj1* DA cKO neurons. We overexpressed VPS35 using a *Cre*-dependent AAV2/5-hSyn-DIO-BFP-VPS35 together with AAV9-hSyn-DIO-pHmS-D2S. Despite variability, VPS35 overexpression restored axonal surface D2S fraction from 30% to an average of 70% in *Synj1* DA cKO neurons, comparable to the control levels (Fig. 7D–E). Consistent with this result, VPS35 overexpression also restored axonal surface DAT-pHluorin fraction in *Synj1* DA cKO neurons, accompanied by an increase in vesicular pH (Fig. 7I, J).

Together, these results show that increasing VPS35 is sufficient to restore presynaptic pHmS-D2S and DAT-pHluorin surface levels despite lipid deficits in Synj1-deficient neurons, indicating that VPS35 availability is rate-limiting for surface trafficking at dopaminergic synapses.

### Synj1 and VPS35 show correlated expression and spatial association in dopaminergic axons.

**Figure 8:**
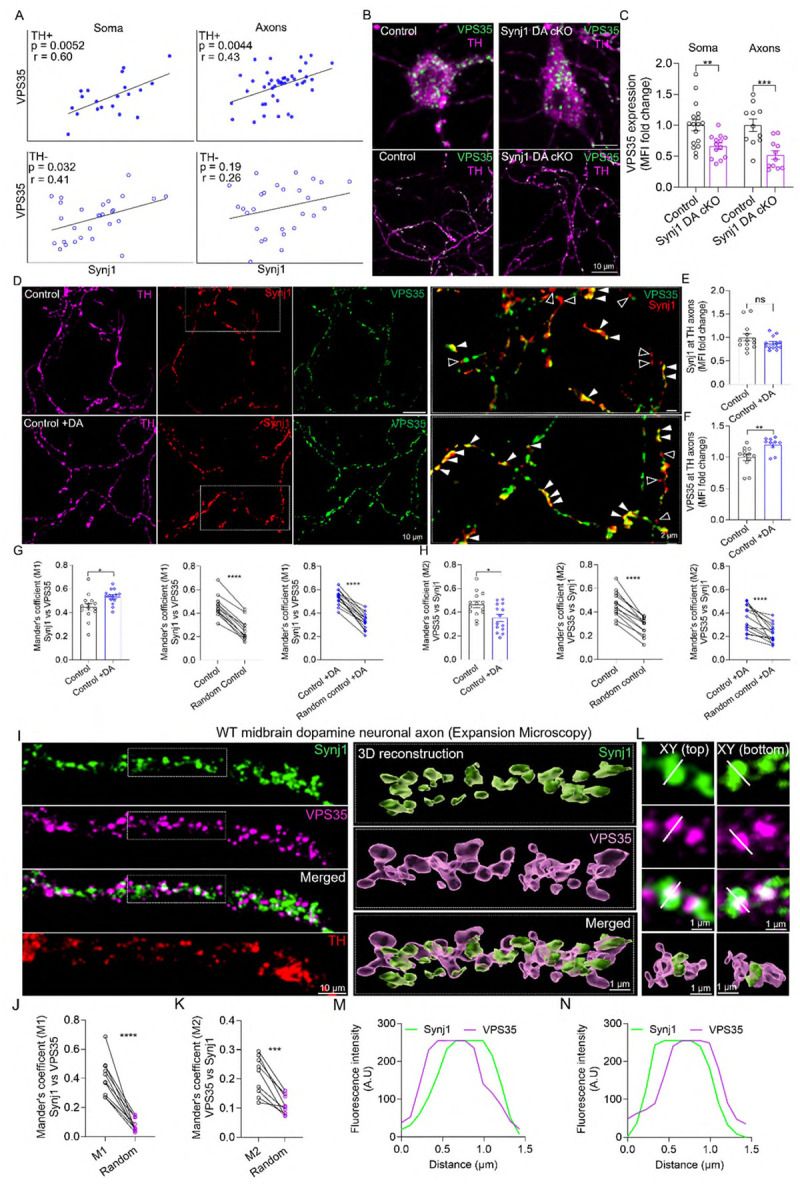
Synj1 and VPS35 exhibit coordinated expression and dynamic spatial colocalization in dopaminergic axons. **A)** Scatter plots showing paired measurements of Synj1 and VPS35 fluorescence intensity from the same soma or axonal regions of TH+ (dopaminergic) and TH- neurons. Linear regression lines are shown with Pearson’s correlation coefficients (r) and corresponding *p* values. Each point represents an individual neuron (soma) or axonal region (TH+ soma n=20, TH- soma n=28, TH*+* axons n=43, TH- axons n=28). Data are from three independent culture preparations. **B)** Representative images of neurons showing VPS35 (green) expression in TH+ (Magenta) soma and axons from control (*Synj1*^flox/flox^) and *Synj1* DA cKO neurons. Scale bar, 10 μm. **C)** Quantification of VPS35 fluorescence intensity in TH+ soma and axons. **D)** Representative confocal images of neuronal axons under baseline conditions and after three repeated treatments with 10 μM dopamine (DA), immunolabeled for Synj1 (red), VPS35 (green), and TH (magenta). Scale bar, 10 μm. Boxed regions are shown at higher magnification. Scale bar, 2 μm. Solid arrowheads indicate Synj1-positive structures containing VPS35; open arrowheads indicate Synj1-positive structures lacking VPS35. **E-F)** Quantification of Synj1 (E) and VPS35 (F) fluorescence intensity in TH+ axons. **G, H)** Mander’s coefficient analysis in control axons following repeated vehicle or 10 μM dopamine treatment. Fraction of Synj1 colocalized with VPS35 (G), and VSP35 colocalized with Synj1 (H). Randomized controls (40 spatial displacements per image) are shown as line plots to estimate colocalization expected by chance. Data are from two independent culture preparations. **I**) Representative expansion microscopy images of TH+ axons co-immunolabeled for Synj1 (green), VPS35 (magenta), and TH (red). Scale bar, 10 μm. A boxed region is rendered in 3D using Imaris. Scale bar, 1 μm. **J, K)** Mander’s overlap coefficient analysis showing the fraction of Synj1 colocalized with VPS35 (J), and VPS35 colocalized with Synj1 (K). Randomized controls (20 spatial displacements per image) are shown as line plots to estimate colocalization expected by chance. **L)** High magnification view of a representative Synj1-VPS35 punctum shown in XY (top and bottom views). Scale bar, 1 μm. **M, N)** Line-intensity profile across the punctum in XY (top, M; bottom, N). Each data point represents axonal analysis within a field of view. Data are from two independent culture preparations. Data are presented as mean ± SEM with individual data points shown. Statistical significance was determined using paired or unpaired two-tailed Student’s *t*-tests, as appropriate. ns, not significant; *p < 0.05, **p<0.01, ***p < 0.001, ****p < 0.0001

Following establishing the Synj1-VPS35 signaling axis for axonal D2S and DAT surface expression, we took an imaging approach to examine whether and how Synj1 and VPS35 interact at the presynaptic terminal. We first examined the expression levels of endogenous Synj1 and VPS35 in cultured ventral midbrain neurons and how they are regulated by dopamine. In mature WT cultures containing both TH-positive and TH-negative cells, the immunofluorescence of Synj1 and VPS35 exhibited a positive correlation in TH-positive neuronal soma (r = 0.6, *p* = 0.0052) and a weaker but significant correlation in TH-positive axons (r = 0.43, *p* = 0.0044) (Fig. 8A). In TH-negative neurons, a modest correlation was observed in the soma (r = 0.41, *p* = 0.0032), whereas no significant correlation was detected in the axons (r = 0.26, *p* = 0.19) (Fig. 8A). Consistent with the co-regulated expression of Synj1 and VPS35 in dopaminergic neurons, the VPS35 levels were reduced in both the soma and axons of *Synj1* DA cKO neurons (Fig. 8B, C). We next examined whether dopamine alters Synj1 and VPS35 levels in dopaminergic axons. In WT neurons, repeated dopamine perfusion within 10 minutes (as described in Fig. 2) increased VPS35 intensity in the axons, while Synj1 levels remained unchanged (Fig. 8D–F). Given the short duration of dopamine perfusion, this increase likely reflects redistribution of existing VPS35 into axonal compartments.

We then sought to determine the spatial organization and proximity between axonal Synj1 and VPS35. Under basal conditions, Synj1 and VPS35 are partially overlapped (M1: 45%, M2: 46%) in WT TH-positive axons, and this organization significantly segregates from random coincidence (Fig. 8G–H). Following repeated dopamine perfusion as described above, the fraction of Synj1-positive compartments containing VPS35 increased by ~10% (Fig. 8G), whereas the fraction of VPS35 within Synj1-positive compartments decreased (Fig. 8H). These data indicate that dopamine promotes recruitment of VPS35 to Synj1-positive sites while also redistributing VPS35 across axonal compartments.

To resolve the spatial relationship between Synj1 and VPS35 at higher resolution, we performed expansion microscopy (ExM, ~4.2×; ~50–70 nm effective resolution) in dopaminergic axons. We found that VPS35-positive structures were frequently positioned within Synj1-positive compartments in TH+ axons (Fig. 8I). Three-dimensional reconstruction confirmed close apposition of VPS35 to these compartments (Fig. 8I). Line-intensity profiles across individual puncta showed that Synj1 and VPS35 signals were largely coincided, with a small but consistent offset at the edges (Fig. 8L, M, N), indicating close spatial association. Consistent with this, Mander’s coefficient showed partial colocalization of Synj1 with VPS35 (M1: 41 %) and VPS35 with Synj1 (M2: 21%), both significantly above randomized controls (Fig. 7J, K). Together, these data show that VPS35 localizes within Synj1-associated axonal compartments in dopaminergic neurons, consistent with their coordinated role in presynaptic sorting.

## Discussion

Synaptic dysfunction is a key feature of early PD, yet the underlying synaptic mechanisms remain poorly understood. Here, we show that the PD-associated Synj1, a presynaptically enriched phosphoinositide phosphatase^40,79–81^, facilitates endosomal sorting of D2S and DAT to regulate dopamine release and maintain presynaptic membrane integrity. Loss of Synj1 leads to endosomal swelling, retention of surface cargo, striatal DAT clustering, reduced dopamine storage, and locomotor deficits. We Further show that dopamine-induced endosomal PI4P remodeling facilitates recruitment of the PD-linked retromer component VPS35, uncovering a convergent signaling pathway linking two PD-associated genes at presynaptic endosomes (Supplemental Fig. S1). VPS35 overexpression rescued presynaptic endosomal sorting in Synj1-deficient neurons despite lipid impairments. We also found corelated expression and co-clustering of Synj1 and VPS35 in dopaminergic axons, supporting their broader impact on surface proteins beyond DAT and D2S, which might be important for axonal maintenance. Together, these findings reveal presynaptic endosomal lipid remodeling as a critical mechanism for sustained dopamine signaling and synaptic integrity. Our data suggests that dopamine release defects often observed in PD models are not necessarily secondary to neurodegeneration. Instead, endosomal stress-induced functional changes prior to substantial axonal degeneration are sufficient to impair dopamine release and drive locomotor deficits at early stage. These findings define a presynaptic mechanism of dysfunction contributing to PD pathogenesis.

Understanding the synaptic mechanisms underlying early PD has led to increasing interest in functional interactions among PD-linked synaptic genes. In previous work, we showed that the *LRRK2 G2019S* mutation exacerbates synaptic vesicle exocytosis defects in *Synj1* haploinsufficient neurons^82^. However, these synergistic synaptic defects were associated with only modest impairments in motor behavior^82^. A recent study of Synj1 and Auxilin double-mutant mice reported enhanced dystrophic changes in the striatum, including widespread DAT clustering and shortened lifespan^24^. Further, Synj1 was previously shown to rescue lipid deficits and neurodegeneration in a *Drosophila* model carrying the PD-associated Auxilin mutation^25^. Here, we demonstrate a Synj1-VPS35 signaling axis regulating endosomal sorting and surface delivery of D2S and DAT. Notably, VPS35 overexpression rescued endosomal sorting defects in Synj1-deleted dopamine neurons. Together, these findings suggest a signaling network among Auxilin, Synj1, and VPS35 in presynaptic lipid regulation and endosomal function. This is further supported by an Endo-PI organelle proteomic study showing Synj1 association with endosomal compartments in human-derived neurons^83^. Our data suggest that Synj1 not only regulates VPS35 spatial organization at presynaptic terminals, including its enrichment in dopaminergic axons and recruitment to presynaptic endosomes, but also maintains VPS35 expression in dopaminergic neurons. Consistent with this, VPS35 levels are substantially reduced in Synj1-deficient dopamine neurons. Although the mechanism underlying their corelated expression remains unclear, Synj1 loss may alter endosomal lipid composition and/or impairs Rab7a-dependent VPS35 stabilization^84^, thereby destabilizing VPS35 and promoting its mislocalization or degradation. VPS35 degradation may be further enhanced by elevated lysosomal proteolytic activity, as previously reported in Synj1-deficient neurons^85^.

Our *in vitro* and *vivo* data supports a model in which Synj1-dependent remodeling of endosomal PI4P regulates molecular organization at presynaptic terminals. Increased endosomal PI4P likely signals the recruitment of a phosphoinositide-interacting sorting nexin (SNX) family protein, facilitating assembly of the VPS35 retromer complex^86^. Indeed, a recent biochemical reconstitution study identified phospholipid composition as a major determinant of retromer and sorting nexin recruitment to endosomal membranes^31^. Previous studies showed that SAC2-mediated hydrolysis of endosomal PI4P is required for endocytic recycling and that deletion of SAC2 impairs endosomal function^72,87^. Conversely, Synj1 overexpressing neurons with enhanced PI4P hydrolysis also exhibit endosomal defects^32^. Together these findings suggest that at least a transient increase in endosomal PI4P is required for presynaptic endosomal sorting. This increase in PI4P is likely mediated by the 5’-phosphatase activity of Synj1 in hydrolyzing endosomal PI(4,5)P_2_^88,89^, or it could also result from the phosphatidylinositol-4 kinase activity in synthesizing PI4P following the SAC1 phosphatase-mediated hydrolysis of endosomal PI3P. Thus, a balanced endosomal lipid metabolism is key to presynaptic endosomal function. Synj1 deletion tips the balance and results in an endosomal traffic jam. Additionally, we found that presynaptic endosomal sorting is regulated by extracellular dopamine. We show that the D2S trafficking defects were only observed in dopaminergic terminals but not non-dopaminergic terminals at baseline. Both VPS35 and Rab7a were recruited to presynaptic D2S containing endosomes following dopamine exposure, and the endosomal PI4P level also exhibits a dopamine-dependent increase in the axons. These findings suggest that tonic dopamine release imposes a sustained lipid metabolic demand at presynaptic terminals that require Synj1 and associated lipid regulatory pathways.

Pathological DAT clusters have previously been reported in the PARK20 model lacking Synj1 SAC1 activity and in Auxilin knockout mice. However, these dystrophic structures were only found in the dorsal but not ventral striatum^24,72,90^. In addition, DAT was previously thought to accumulate at the plasma membrane^24,72^. Here, we provide evidence that dystrophic DAT clusters are trapped in early endosomes in Synj1-depleted dopamine neurons. We further show that the volume of endosomes associated with dystrophic DAT clusters increased nearly 4-fold in the dorsal striatum, compared to a 2-fold increase in the ventral striatum, suggesting a better coping strategy in the less vulnerable part of the brain. As the *Synj1* DA cKO mice (lacking both SAC1 5’-phosphatase enzymes) exhibited DAT clusters throughout the striatum, it is possible that the 5’-phosphatase activity is specifically responsible for maintaining endosomal and DAT functions in the ventral striatum.

Taken together, our findings show that Synj1 integrates lipid signaling with retromer-dependent endosomal cargo sorting, a process essential for axonal membrane quality control. Beyond regulating presynaptic surface D2S and DAT availability, additional surface cargos controlled by this signaling pathway remain to be identified. A previous study reported reduced transferrin receptor levels in Synj1-deficient cells^33^. Given the central role of transferrin receptors in transporting iron for lysosomal and mitochondrial function, it would be intriguing to find out whether the Synj1-VPS35 signaling axis contributes to iron homeostasis and iron-associated oxidative stress. Notably, our data showed that increasing VPS35 levels was sufficient to overcome the endosomal lipid defect in *Synj1*-null neurons, suggesting that VPS35 recruitment is the rate-limiting step in presynaptic surface cargo delivery. VPS35-based therapeutic strategies have been proposed and shown to be effective in various models of neurodegenerative disorders^91,92^. Whether it can be used for correcting the surface proteome of dopaminergic neuron during early PD pathogenesis awaits further investigation.

## Materials and Methods

### Ethical approval

All animal studies were conducted in accordance with the National Institutes of Health (NIH) and with protocols (PROTO201800183) approved by the Institutional Animal Care and Use committee (IACUC) of Rutgers University.

### Animals

Mice were housed under normal 12 h day/night cycle in a pathogen-free barrier facility at the Rutgers Robert Wood Johnson Medical School Research Tower vivarium. The *Synj1* HET mouse was originally gifted by the Pietro De Camili laboratory at Yale University. The *C57BL/6J* mice were purchased from the Jackson laboratory. *C57BL/6J* mice were crossed with *Synj1* HET mice to generate littermate mice for midbrain culture and for behavioral studies. The number of mice used in each behaviors test is included in figures and figure legend. The Synj1 condition ready allele (Tm1c) was generated by MRC Harwell (UK) with *loxP* sites flanking the critical exon 4. *Synj1*^flox/flox^ mice were identified using 5arm-WTF and Crit-WTR primers, which produces a single band that migrates at ~550 bp. *Synj1*^flox/flox^ mice was crossed with heterozygous *DAT*^*IRESCre*^ (*DAT-Cre*) mice (Jackson Laboratory, stock# 06660) for two generations to obtain the *DAT-Cre(+/−);Synj1*^flox/flox^ or *Synj1* DA cKO mice. These mice were viable and fertile and the conditional deletion of *Synj1* in ventral midbrain dopamine neurons has been confirmed. The *Synj1* DA cKO mice were crossed with *Synj1*^flox/flox^ mice to generate 50% *Synj1* DA cKO pups following mendelian inheritance. The *DAT-Cre* cultures were prepared separately by crossing *DAT-Cre* HET with *C57BL/6J* mice. This breeding strategy allows enough pups born in a litter with the desired genotype for pooled ventral midbrain culture containing pups of both sexes.

### Antibodies

The following primary antibodies were used in this study: polyclonal anti-guinea pig Synapsin I/II (Synaptic System, Cat. #106004, 1:500), polyclonal anti-goat VPS35 (Novus Biologicals, Cat. #NB100–1397; 1:500), rabbit anti-TH (Novus Biologicals, Cat. #NB300–109, 1:1000), mouse anti-TH (Sigma, Cat. #T2928, 1:1000), polyclonal chicken anti-TH (Sigma-Aldrich, Cat. #AB9702), rat anti-DAT (Millipore, Cat. #MAB369; 1:500), polyclonal anti-rabbit Synj1 (Cat. #NBP1–87842; 1:500), monoclonal mouse anti-FLAG (Sigma-Aldrich, Cat. #F1804; 1:500), polyclonal rabbit anti-Flag (Proteintech, Cat. #20543–1-AP; 1:500), purified Anti-PtdIns(4)P IgM (Echelon Bioscience, Cat. #Z-P004; 1:250), rabbit monoclonal EEA1 (Abcam, Cat. #ab109110; 1:500), anti-rab7a (Abcam, Cat. #ab50533). Secondary antibodies: Donkey anti-mouse Alexa Fluor 488 (Invitrogen, Cat. #A-21202; 1:1000), donkey anti-rabbit Alexa Fluor 555 (Invitrogen, Cat. #A-31572; 1:1000), donkey anti-chicken Alexa fluor 488 (Invitrogen, Cat. #A78948; 1:1000), donkey anti-goat Alexa Fluor 647 (Invitrogen, Cat. #A-21447; 1:1000), Donkey Anti-Goat IgG ATTO647N (Hypermol, Cat. #2910; 1:250).

### Solutions and buffers

All solutions were prepared using analytical-grade reagents (Sigma-Aldrich, unless otherwise noted) and adjusted to the appropriate pH using NaOH or HCl.

Tyrode’s solution (pH 7.40): 30 mM Glucose, 25 mM HEPES, 2.5 mM KCl, 119 mM NaCl, 10 μM 6-cyano-7- nitroquinoxaline-2,3-dione (CNQX), 2 mM MgCl_2_, 2 mM CaCl_2_ and 50 μM D,L-AP-5. The MES buffer: (pH 5.50): 25 mM MES, 30 mM D-glucose, 70 mM NaCl, 2 mM MgCl_2_, 2.5 mM KCl, 2 mM CaCl_2_, 50 μM AP-5, 10 μM CNQX, and buffered to pH 5.50. NH_4_Cl buffer: (pH 7.40): 50 mM NH_4_Cl, 30 mM Glucose, 2.5 mM KCl, 2 mM MgCl_2_, 70 mM NaCl, 2 mM CaCl_2_, 25 mM HEPES, 50 μM AP-5 and 10 μM CNQX.

### Constructs and virus

The pCAGP-FLAG-pHmScarlet/pHluorin-hD2S were engineered using a previously published pCAGP-hDAT-pHluorin backbone^93^. The pHmScarlet cDNA was amplified from VAMP2-pHmScarlet (Addgene, Cat. #166890). The following linkers were added on the 5’ and 3’ of the pHmScarlet, respectively: TGSTSGGSGGTGG and SGGTGGSGGTGGSGGTG. The linker-pHmScarlet-linker sequence was subcloned into pcDNA-FLAG-hD2S-L-Venus (Addgene, Cat. #19966) via an inserted Age-I restriction site immediately following a signal peptide (MKTIIALSYIFCLVFA) and a FLAG sequence. The open reading frame (ORF) containing signal peptide-FLAG-linker-pHmScarlet-linker-D2S was then subcloned into the pCAG backbone vector replacing the hDAT-pHluorin. The ORF was validated by sequencing before use. The same ORF was synthesized into a pAAV9-hSYNp-DIO vector by commercial service, VectorBuilder and packaged into high titer (>10e13 GC/mL) AAV9 particles for transduction of *DAT-Cre* and *Synj1* DA cKO cultures. The pDEST-eGFP-C1-VPS35 was purchased from Addgene (Cat. #163622). The AAV2/5-hSYNp-DIO-BFP-hVPS35-tWPA was synthesized and packaged at high titer (>10e12 GC/mL) by OBiO Inc. pRP-TagBFP2-U6>mVPS35 shRNA was designed and synthesized by VectorBuilder using the following targeting sequence: AGCTTAACCTTGAACATATTG.

### Neuronal culture, transfection and AAV transduction

Primary ventral midbrains (MB) cultures were prepared from postnatal day 0–1 (P0-P1) WT, *Synj1* HET, *DAT-Cre* HET and *Synj1* DA cKO mouse pups as previously described^64,94,95^. The genotype of each pup was determined by PCR and at least 2~3 pups in the same genotype were pooled to prepare the culture. As described before^94^, MB tissue containing the substantia nigra (SN) and ventral tegmental area (VTA) was dissected and digested using papain (Worthington, Cat. #LK003178) in a 34–37°C constant oxygenated water bath with gentle stirring. Dissociated neurons were then seeded at a density of 3 × 10^4^ cells per 0.28 cm^2^ area within cloning cylinders placed on poly-L-ornithine-coated coverslips (Sigma, Cat. #P3655). Cells were maintained in Neurobasal-A medium (Gibco, Cat. #12349015) supplemented with 10 ng/mL glial cell-derived neurotrophic factor (GDNF; EMD Millipore, Cat. #GF030).

At DIV 5–7, neurons were transfected with the indicated plasmid DNA using Lipofectamine 2000 (ThermoFisher, Cat. #11668019) as per the manufacturer’s instructions with minor modifications. After 45 min of incubation at 37°C, the transfection mixture was washed off with 1 × MEM, and cultures were returned to fresh medium containing GDNF and cytosine β-arabinofuranoside (Ara-C) to suppress glial proliferation. Imaging experiments were conducted between DIV 13 and DIV 17.

For viral transduction, 1 μL of the high titer AAV9 or AAV2/5 particles were added directly onto the neuronal culture grown within the cylinder on DIV 3. After 3 days of incubation, a full media change was conducted, and cylinders were removed until imaging experiments on and after DIV 13. All procedures were in accordance with institutional guidelines on handling hazardous materials and waste management.

### Sniffer cell culture and co-culture with midbrain neurons

GRAB_DA2m_-expressing sniffer cells^68^ were maintained in DMEM (ThermoFisher Cat. #11965092) medium supplemented with 10% fetal bovine serum (Atlanta Biologicals, Cat. #S11550H), 1% Pen/Step solution (Thermo Fisher, Cat. #15140122), 15 μg/mL Blasticidin (Millipore Sigma, Cat. #15205), and 200 μg/mL Hygromycin (Millipore Sigma, Cat. #H3274). Cells were cultured in a humidified incubator at 37°C with 5% CO_2_.

For co-culture experiments, sniffer cells were seeded at a density of 20,000 cells per 8 × 8 cloning cylinders onto DIV 13 midbrain neuronal cultures in neuronal medium. After overnight incubation, doxycycline (1 μg/mL) was added to induce GRAB_DA2m_ expression for 24 h. Imaging was performed at DIV 15. Field electrical stimulation was applied using a custom-built stimulation chamber with two platinum electrodes. 1 ms square pulses were generated and delivered by an A310 Accupulser and A385 stimulus isolator (World Precision Instruments) to evoke action potential. Dopaminergic axons were determined blindly based on sniffer cell responses and verified *post hoc* by immunofluorescence against TH and Synapsin I/II.

### Live-cell imaging of pHmscarlet-D2S in cultured neurons

Live imaging of pHm-D2S-expressing midbrain neurons was performed in custom-designed laminar-flow chamber equipped with continuous gravity perfusion, maintained at ~30°C. Cells were initially perfused with Tyrode’s solution at a rate of 0.2–0.4 mL/min. For pH-sensitive fluorescence measurements, MES buffer (pH 5.50) was perfused to quench surface-localized fluorescence, and NH_4_Cl buffer (pH 7.40) was applied to neutralize intracellular acidic compartments and reveal the total D2S fluorescence. Imaging was performed using our Nikon Ti2 wide-field inverted microscope equipped with 60× oil-immersion objective and a back-illuminated EM-CCD camera (Andor iXon + DU-897). Images were acquired at 1 frame per second using NIS-element software. Neuronal positions were saved according to a reference point for *post hoc* immunofluorescence analysis.

### Immunofluorescence

Primary midbrain neurons were seeded on poly-L-ornithine-coated coverslips as described above. For experiments involving dopamine treatment, neurons were perfused with freshly prepared 10 μM dopamine in Tyrode’s solution for 3-min pulses, each followed by a 3-min wash with Tyrodes’ solution. Immediately after the final dopamine perfusion, cells were fixed in pre-warmed 4% paraformaldehyde (PFA) for 10 min at room temperature (RT) followed by PBS washes. Cells were permeabilized with 0.2% Triton X-100 diluted in PBS for 15 min at RT, washed three times in PBS, and blocked in 5% bovine serum albumin (BSA, w/v) for 45 min. Following blocking, cells were incubated overnight at 4°C with primary antibodies against VPS35, synj1, and tyrosine hydroxylase (TH), diluted in blocking buffer. The next day, cells were washed in PBS and incubated with fluorophore-conjugated secondary antibodies for 1 hour at RT. After washing, coverslips were mounted using Clear-Mount solution (Invitrogen, Cat. #00-8-10) and stored in the dark until imaging.

For surface and intracellular FLAG-D2S immunostaining, neurons transfected with FLAG-D2S were fixed with 4% PFA for 10 min at RT, followed by washed three times with PBS. Surface FLAG staining was performed by blocking the cells in 5% BSA for 45 min at RT, then incubating with rabbit anti-FLAG primary antibody (1:500) diluted in blocking buffer overnight at 4°C. After washing, cells were incubated with fluorophore-conjugated secondary antibody for 1 hour at RT. Following final washes cells were processed for intracellular labeling. For intracellular FLAG detection, neurons were permeabilized with 0.2% Triton X-100 diluted in PBS for 15 min, re-blocked in 5% BSA for 45 min. Cells were then incubated overnight at 4°C with a second mouse anti-FLAG primary antibody (1:500), together with either anti-TH (1:1000), anti-VPS35 (1:500), or Anti-RFP (1:500, for Rab7a detection), diluted in blocking solution. After washing with PBS, appropriate fluorophore-conjugated secondary antibodies were applied for 1 h at RT. Finally, cells were washed, and coverslips were mounted onto slides using Clear-mount solution. Images were acquired using our confocal microscopy.

### Confocal imaging

Images for localization analysis were acquired using a Nikon CREST spinning disk confocal microscope. The system includes laser lines at 405 nm, 488 nm, 561 nm, 647 nm, and 701 nm for multi-channel fluorescence imaging. A 100 X oil-immersion objective was used for all acquisition. Laser power, exposure time, and binnings were kept constant within each experimental group. Z-stacks were collected at 0.5–0.7 μm step using Nikon Elements software.

### Confocal microscopy image analysis

All image analyses were performed using Fiji (ImageJ). For Synj1 and VPS35 colocalization in axons, maximum intensity projections were generated from confocal z-stacks images. Background subtraction was performed using the rolling ball algorithm (radius = 50). Axonal regions of interest (ROIs) were manually delineated by using TH-positive signal as a guide. Manual intensity thresholds were applied separately to each channel and held constant across conditions. Colocalization analysis was carried out using the BIOP JACoP Plugin to calculate Mander’s overlap coefficients (M1 and M2), measuring the fraction of signal in one channel overlapping with the other within axonal ROIs.

To control for random spatial coincidence, we implemented a lateral shift-based randomization strategy as previously described^96–98^, with slight modifications for axon specific analysis. A custom-written Fiji macro was used to perform 40 independent lateral displacements of the VPS35 channel relative to the fixed Synj1 channel, each involving unique x- and y -axis shifts that preserved signal structure but disrupted spatial alignment. Mander’s overlap coefficients (M1 and M2) were recalculated for each randomized configuration using the JACop, and the average of these 40 values used for comparison. This was then compared to the unshifted (true) Mander’s coefficient obtained from the original, aligned images. For each experimental condition, 14 images were analyzed independently, with each image contributing a signal data point derived from the average for 40 randomized values. Statistical significance between true and randomized colocalization was assessed using a paired, two-tailed t-test.

Expression levels of VPS35 and Synj1 in TH+ axons and soma were quantified using Fiji. Background subtraction was performed using the rolling ball algorithm. TH+ axons were identified by thresholding the TH channel and converting it into a binary mask using the “create selection” function. These same masks were applied to the VPS35 and Synj1 channels, and mean fluorescence intensity was measured within the masked axonal regions. For soma analysis, ROIs were manually outlined using the freehand tool based on TH signal. As a validation step, an alternative approach was used in which ROIs were manually drawn, and background-subtracted mean intensities were calculated by measuring signal from nearby non-axonal regions. Both approaches yielded comparable results. Only values obtained using the rolling ball-corrected analysis were used in the final quantification and statistical comparisons. For representative images shown in figures, the smooth filter in Fiji was applied to minimize pixel noise and improve visualization.

### PI4P immunostaining and image analysis in primary midbrain neuronal cultures

Neurons were subjected to repeated dopamine perfusion as described above prior to fixation. Immunostaining for endosomal PI4P was performed following the manufacturer’s protocol (Echelon Biosciences) and previous reports^34,99^, with minor modifications. All steps were performed in room temperature. Cells were fixed in 2% formaldehyde (FA) in PBS for 15 minutes, and then cells were rinses three time PBS containing 50 mM NH_4_Cl. Cells were permeabilized with Buffer 1 (20 mM PIPES, 137 mM NaCl, 2.7 KCl, pH 6.8) containing 0.1% saponin for 5 min, washed there times with buffer 1, and blocked in buffer 1 supplemented with 5% (v/v) normal goat serum and 50 mM NH_4_CL for 45 min. Primary antibody against PI4P (1:200), EEA1 (1:500), and Synapsin (1:500) were diluted in buffer 2 (buffer 1 + 5% (v/v) normal goat serum) incubated for 1 h. Cells were then washed twice in buffer 1. Secondary antibodies were diluted in buffer 2 and incubated for 1 h. Cells were washed four times with buffer 1 and post-fixed in 2% FA in PBS for 10 min. After post-fixation, cells were rinsed three times with 50 mM NH_4_Cl in PBS, and once with distilled water (Corning, Cat. # 25–055-CV). Coverslips were mounted using mounting media and stored at 4°C in the dark until imaging.

Image analysis was performed using ImageJ (Fiji) and Imaris Bitplane (v 11.01). EEA1-positive endosomes were segmented using the surface detection module in Imaris with fixed thresholding applied across all samples. Synapsin-positive axonal regions, including continuous axonal structures and Synapsin-enriched puncta, were also segmented to define presynaptic compartments. EEA1 surfaces were used to generate masks, and PI4P intensity was quantified within EEA1-positive endosomal masks restricted to Synapsin-positive axonal compartments, including both axonal shafts and Synapsin-enriched presynaptic puncta. Axonal PI4P levels were independently quantified in Fiji by measuring mean fluorescence intensity within Synapsin-positive axonal ROIs. For each field of view, mean PI4P intensity was calculated and averaged to generate a single value per field, and multiple fields were analysed per conditions. Experiments were performed using two independent primary midbrain neuronal cultures derived from separate litters. For each culture, two technical replicates were included per conditions.

### VPS35 enrichment in EEA1-positive endosomes in Neuro-2a (N2A) cells

N2A cells were transfected with GFP and D2S-Flag (control) or SAC2-GFP and D2S-flag and subjected to repeated dopamine perfusion or left untreated, as indicated. Following treatment, cells were fixed in 4% PFA in PBS for 15 min at room temperature and washed three times with PBS. Cells were permeabilized with 0.1% saponin in PBS for 5 min and blocked in PBS containing 5% BSA for 45 min. Cells were incubated with primary antibodies against GFP (1:1000) VPS35 (1:500) and EEA1 (1:500) diluted in 5% BSA overnight in 4°C, followed by three washes with PBS. Then cells were incubated in appropriate secondary antibodies (1:1000) diluted in 5% BSA for 1 h at room temperature. Cells were washed three times with PBS, 5 min each time. Coverslips were mounted using mounting media and stored at 4°C in the dark until imaging.

Image analysis was performed using Fiji. Only transfected cells (GFP-positive or SAC2-GFP positive) were used for analysis. ROIs corresponding to individual transfected cells were manually defined based on GFP signal and used for subsequent quantification. EEA1-positive endosomes were identified by thresholding the EEA1 channel, followed by particle analysis to detect discrete endosomal structures. Size and Circularity parameters were defined based on control samples and applied uniformly across all conditions. ROIs corresponding to individual EEA1-positive particles were used to measure VPS35 fluorescence intensity. VPS35 intensity was averaged across EEA1-positive endosomes within each cell to obtain a single per cell value. Per-cell values from multiple cells within each field of view were averaged to obtain a single value per field, which was used as the unit for statistical analysis across conditions. Multiple fields of view were analysed per condition. Experiments were performed in two independent biological replicates, each comprising two technical replicates per condition.

### Expansion microscopy (ExM)

Following immunostaining, samples were processed for expansion according to the published protocol^100^ with minor modifications. Samples were incubated overnight at room temperature in 0.1 mg/ml Acryloyl-X SE (AcX; Invitrogen, Cat. #A20770) prepared by diluting a 10 mg/mL AcX/DMSO stock 1:100 into 1× PBS. The next day, samples were washed two times in 1× PBS for 15 min each.

#### Gelation:

A monomer stock solution (Stock X) was prepared containing 4 M sodium acrylate (Sigma, Cat. #408220), 7M acrylamide (Sigma, Cat. #A9099), 130 mM N, N’-methylenebisacrylamide (Sigma, Cat. #146072), and 5 M sodium chloride in 1× PBS. Immediately before embedding, Stock X was mixed with ddH_2_O, 10% TEMED, and 10% APS at a 47:1:1:1 (V/V) ratio to prepare the gelling solution. Samples were placed in custom gelation chambers, filled with this mixture, and polymerized at 37°C for 1 h.

#### Digestion:

After polymerization, gels were incubated in digestion buffer containing 0.5% Triton X-100, 1 mM EDTA (pH 8), 50 mM Tris-HCl (pH 8), and 800 mM sodium chloride, supplemented with proteinase K (NEB, Cat. #P8107S) at a final concertation of 8 U/mL. Digestion was carried out in dark for 2 h at room temperature.

#### Gel expansion and expansion factor (EF) calculation:

Gels were expanded by three sequential washes in ultrapure water (20 min each) until reaching stable size. To calculate the EF, gels were imaged with a ruler placed adjacent to them before and after expansion. Gel dimensions were measured in Fiji, and EF was calculated as post-expansion gel length divided by the pre-expansion gel lengths. Measurements were taken at multiple positions along each gel and averaged to yield a single EF per gel. Across replicates, the mean linear EF was ~4.2×.

#### Imaging:

Expanded gels were mounted cell-side down on poly-D-lysine-coated coverslips (1 mg/mL, coated overnight, and rinsed in water) within a standard imaging chamber and secured with a custom holder. Ultrapure water was added to fully cover the gel and prevent drying or shrinkage during imaging. Post-expansion imaging was performed on a Nikon CREST spinning-disk confocal microscope equipped with a 100× oil-immersion objective (Plan Apo λ, NA 1.45, n = 1.515) using a fixed pinhole array of 50 μm, equivalent to ~1 Airy unit. Excitation/emission settings were 477/510 nm for Synj1 (Alexa Fluor 488), 546/595 nm for TH (Alexa Fluor 555), and 638/685 nm for VPS35 (Atto 647). Images were acquired at 0.11 μm/pixel (1200 × 1200) and 0.7 μm Z-steps. Diffraction-limited resolutions were estimated as 0.61 λ / N A (lateral) and 1.4 n λ / NA^2^ (axial), corresponding to ~215/515 nm (XY/Z) at 510 nm, ~250/600 nm at 595, and ~288/691 nm at 685 nm. According to Nyquist sampling criteria, the target sampling is ~0.11 μm (510 nm), ~0.125 μm (595 nm), and ~0.144 μm (685 nm) in XY and ~0.26 μm (510 nm), ~0.30 μm (595 nm) and ~0.35 μm (685 nm) in Z. With a measured 4.2 × linear expansion, the effective sampling improves to ~0.026 μm/pixel (XY) and ~0.167 μm (Z), and the effective diffraction-limited lateral resolution (XY/4.3) is ~51 nm (510 nm), ~60 nm (595 nm), and ~69 nm (685 nm).

#### ExM image analysis:

Images were analysed using Fiji (ImageJ) and representative 3D rendering were generated using Imaris (Version 10). Background was subtracted using a rolling ball algorithm (radius = 50). Axonal regions of interest (ROIs) were manually delineated by using TH-positive labelling along with Synj1-positive signal as a guide. Colocalization analysis was performed with the BIOP JACoP Plugin to calculate Mander’s overlap coefficients (M1 and M2). Intensity thresholds were set manually for each channel and then held constant across conditions. To control for random spatial coincidence, we implemented a lateral shift-based randomization strategy as described above. A custom Fiji macro was used to perform a minimum of 20 independent lateral displacements of the VPS35 channel relative to the fixed Synj1 channel, each involving unique x- and y -axis shifts that preserved signal structure but disrupted spatial alignment. Mander’s coefficients were recalculated for each randomized configuration in JACoP, and the average of these 20 randomized values was compared with the unshift (true) coefficient from the original aligned images. For each experimental condition, 9 images were analyzed independently, with each image contributing a signal data point derived from the average for 20 randomized values. Statistical significance between true and randomized colocalization was determined using t-test.

### Behavioral tests

All behavioral experiments were conducted during daytime under ambient light following 1 h acclimation to the testing environment. Mice were randomly assigned to experimental groups. All testing apparatuses were thoroughly cleaned between animals.

#### Open field test

Locomotion activity was assessed using the VersaMax Legacy Open Field system (40 × 40 × 30 cm). Mice were placed individually in the central of the square arena and allowed to explore freely for 30 min. Total distance travelled and mean velocity were quantified using automated tracking software Fusion.

#### Accelerated rotarod test

Motor coordination and balance were evaluated using an accelerating rotarod apparatus. Prior to testing, mice were trained on the rotarod at a constant speed of 25 rpm for 5 min (three trials), with at least 10 min rest between trials. Mice were tested in cohorts of five by placing them on the rotarod starting at 4 rpm, with speed gradually increasing to 40 rpm over 2.5 min and maintained at 40 rpm for the remainder of the trial. Latency to fall was recorded for each mouse. The average of three trials for each mouse was used for analysis.

#### Wire hang test

Muscle strength of mice was measured using a wire hang test. Mice were placed on a wire grid, which was then gently inverted to allow the mice to hang upside down, with soft bedding below to prevent injury upon falling. Each mouse completed three trials and average of the trials were used for analysis

#### Balance beam test

Mice were trained for 3 consecutive days to traverse a three-foot 2 cm-wide beam to its home cage. One the fourth day, mice were tested on a 0.5 cm-wide beam by placing them at one end and allowing them to traverse to the opposite end. The number of foot slips and traversal time were videotaped for each trial. Each mouse completed three trials with at least 10 min rest between trials. The mean number of foot slips and traversal time across trials for each mouse were used for analysis.

#### Quinpirole-induced locomotor suppression

Locomotor activity was used to assess D2 receptor pharmacosensitivity *in vivo*. Two cohorts of mice containing both male and females were randomly assigned to receive intraperitoneal injections of either saline or quinpirole (0.5 mg/kg, Tocris Bioscience, Cat. #1061), freshly prepared in sterile saline. Immediately following injection, mice were placed into the center of a clean open field arena (19 ×19 inches) equipped with a VersaMax monitoring system and allowed to explore freely for 45 min. Total distance travelled was quantified over the testing period.

### Immunohistochemistry

Mice were deeply anesthetized with isoflurane and transcardially perfused with 1 x phosphate buffer saline (PBS, 50 mL), followed by 4% paraformaldehyde (PFA, 50 mL) in PBS. Brains were collected, post-fixed in 4% PFA at 4°C, and cryoprotected in increasing concentrations of sucrose (10%, 20% and 30% in PBS) until they sank. Brain tissues were embedded in embedding medium and sectioned at 40 μm thickness using a cryostat (Leica cryostat CM1900).

Immunostaining was performed on free-floating sections. Sections were washed in PBS three times, 5 min each, and then blocking in 10% normal goat serum in PBS containing 1% Triton X-100 for 1 h at room temperature. Sections were then incubated with primary antibodies diluted in blocking solution overnight at 4°C. After washing with PBS three times, 10 min each, sections were incubated with appropriate secondary antibodies for 1 h at room temperature. Sections were then washed in PBS three times, 5 min each, and were mounted onto glass slides, and coverslipped using antifade mounting media. Images were acquired using our spinning disk confocal microscope and analysed with Fiji.

### HPLC measurement of dopamine and its metabolites

Striatal dopamine and its metabolites were measured using high-performance liquid chromatography (HPLC). Mice were anesthetized with isoflurane and euthanized by cervical dislocation. Brains were rapidly removed and placed on ice. The striatum was dissected, flash-frozen in liquid nitrogen, and stored at −80°C until analysis. Tissue samples were sent for preparation and HPLC analysis at the Vanderbilt University Neurochemistry Core. Dopamine, DOPAC, HVA and 3-MT were separated and detected using electrochemical detection.

### Statistical analysis and reproducibility

All statistical analyses were performed using OriginLab or GraphPad Prism (v10-v11). Unless specified otherwise, experiments were independently repeated at least two times using neuronal cultures derived from a minimum of two separate litters. Normality of the data was assessed using the Shapiro-Wilk test or the Kolmogorov-Smirnov test. Outliers were identified using the ROUT method (Q=1%, GraphPad Prism) and excluded prior to statistical analysis. Correlations between two variables were analyzed using Pearson correlation unless otherwise specified. Two tailed Student’s *t*-tests or Mann-Whitney non-parametric tests were used for comparisons between two groups. One-way or two-way ANOVA with appropriate *post hoc* corrections was used for multiple group comparisons. Biological replicates and statistical details including n values, tests, and *p* values are provided in the figure legends. Mice were randomly assigned to experimental groups. Experimenters were not blinded to genotypes during data collection, and data analysis was performed using predefined criteria. Data are presented as mean ± SEM and *p* value < 0.05 was considered statistically significant.

Figures were prepared using Adobe Illustrator and Photoshop (Adobe Inc., San Jose, CA, USA). Schematic illustrations were created using BioRender.com.

## Supplementary Material

1

## Data Availability

All data supporting the findings of this study are presented in the main figures and provided as supplemental materials. Datasets for generating all graphs are deposited into FigShare repository. Raw images and videos are available upon request.

## References

[1] Soukup S. F., Vanhauwaert R. & Verstreken P. Parkinson’s disease: convergence on synaptic homeostasis. EMBO J 37 (2018). 10.15252/embj.201898960 PMC6138432 30065071

[2] Cramb K. M. L., Beccano-Kelly D., Cragg S. J. & Wade-Martins R. Impaired dopamine release in Parkinson’s disease. Brain 146, 3117–3132 (2023). 10.1093/brain/awad064 36864664 PMC10393405

[3] Leonard H. L. & Global Parkinson’s Genetics P. Novel Parkinson’s Disease Genetic Risk Factors Within and Across European Populations. medRxiv (2025). 10.1101/2025.03.14.24319455

[4] Masotti B., Tombesi G., Parisiadou L. & Greggio E. LRRK2 and the fragile synapse: a molecular prelude to Parkinson’s disease? Biochem J 482, 1585–1605 (2025). 10.1042/BCJ20253351 41148193 PMC12687443

[5] Gcwensa N. Z., Russell D. L., Cowell R. M. & Volpicelli-Daley L. A. Molecular Mechanisms Underlying Synaptic and Axon Degeneration in Parkinson’s Disease. Front Cell Neurosci 15, 626128 (2021). 10.3389/fncel.2021.626128 33737866 PMC7960781

[6] Zou L., Tian Y. & Zhang Z. Dysfunction of Synaptic Vesicle Endocytosis in Parkinson’s Disease. Front Integr Neurosci 15, 619160 (2021). 10.3389/fnint.2021.619160 34093144 PMC8172812

[7] Cohen L. D. & Ziv N. E. Recent insights on principles of synaptic protein degradation. F1000Res 6, 675 (2017). 10.12688/f1000research.10599.1 28620464 PMC5461898

[8] Lepeta K. Synaptopathies: synaptic dysfunction in neurological disorders - A review from students to students. J Neurochem 138, 785–805 (2016). 10.1111/jnc.13713 27333343 PMC5095804

[9] Martinez G., Khatiwada S., Costa-Mattioli M. & Hetz C. ER Proteostasis Control of Neuronal Physiology and Synaptic Function. Trends Neurosci 41, 610–624 (2018). 10.1016/j.tins.2018.05.009 29945734 PMC7268632

[10] Nachman E. & Verstreken P. Synaptic proteostasis in Parkinson’s disease. Curr Opin Neurobiol 72, 72–79 (2022). 10.1016/j.conb.2021.09.001 34653835

[11] Edvardson S. A deleterious mutation in DNAJC6 encoding the neuronal-specific clathrin-uncoating co-chaperone auxilin, is associated with juvenile parkinsonism. PLoS One 7, e36458 (2012). 10.1371/journal.pone.0036458 22563501 PMC3341348

[12] Vidyadhara D. J. Dopamine transporter and synaptic vesicle sorting defects underlie auxilin-associated Parkinson’s disease. Cell Rep 42, 112231 (2023). 10.1016/j.celrep.2023.112231 36920906 PMC10127800

[13] Klumperman J. & Raposo G. The complex ultrastructure of the endolysosomal system. Cold Spring Harb Perspect Biol 6, a016857 (2014). 10.1101/cshperspect.a016857 24851870 PMC4176003

[14] Dong J. Endosomal traffic disorders: a driving force behind neurodegenerative diseases. Transl Neurodegener 13, 66 (2024). 10.1186/s40035-024-00460-7 39716330 PMC11667944

[15] Herman M., Randall G. W., Spiegel J. L., Maldonado D. J. & Simoes S. Endo-lysosomal dysfunction in neurodegenerative diseases: opinion on current progress and future direction in the use of exosomes as biomarkers. Philos Trans R Soc Lond B Biol Sci 379, 20220387 (2024). 10.1098/rstb.2022.0387 38368936 PMC10874701

[16] Hurley J. H., Coyne A. N., Miaczynska M. & Stenmark H. The expanding repertoire of ESCRT functions in cell biology and disease. Nature 642, 877–888 (2025). 10.1038/s41586-025-08950-y 40562928

[17] Vazquez-Velez G. E. & Zoghbi H. Y. Parkinson’s Disease Genetics and Pathophysiology. Annu Rev Neurosci 44, 87–108 (2021). 10.1146/annurev-neuro-100720-034518 34236893

[18] Williams E. T., Chen X., Otero P. A. & Moore D. J. Understanding the contributions of VPS35 and the retromer in neurodegenerative disease. Neurobiol Dis 170, 105768 (2022). 10.1016/j.nbd.2022.105768 35588987 PMC9233057

[19] Kirola L., Behari M., Shishir C. & Thelma B. K. Identification of a novel homozygous mutation Arg459Pro in SYNJ1 gene of an Indian family with autosomal recessive juvenile Parkinsonism. Parkinsonism Relat Disord 31, 124–128 (2016). 10.1016/j.parkreldis.2016.07.014 27496670

[20] Krebs C. E. The Sac1 domain of SYNJ1 identified mutated in a family with early-onset progressive Parkinsonism with generalized seizures. Hum Mutat 34, 1200–1207 (2013). 10.1002/humu.22372 23804563 PMC3790461

[21] Lesage S. Clinical Variability of SYNJ1-Associated Early-Onset Parkinsonism. Front Neurol 12, 648457 (2021). 10.3389/fneur.2021.648457 33841314 PMC8027075

[22] Olgiati S. PARK20 caused by SYNJ1 homozygous Arg258Gln mutation in a new Italian family. Neurogenetics 15, 183–188 (2014). 10.1007/s10048-014-0406-0 24816432

[23] Quadri M. Mutation in the SYNJ1 gene associated with autosomal recessive, early-onset Parkinsonism. Hum Mutat 34, 1208–1215 (2013). 10.1002/humu.22373 23804577

[24] Ng X. Y. Mutations in Parkinsonism-linked endocytic proteins synaptojanin1 and auxilin have synergistic effects on dopaminergic axonal pathology. NPJ Parkinsons Dis 9, 26 (2023). 10.1038/s41531-023-00465-5 36792618 PMC9932162

[25] Jacquemyn J. Parkinsonism mutations in DNAJC6 cause lipid defects and neurodegeneration that are rescued by Synj1. NPJ Parkinsons Dis 9, 19 (2023). 10.1038/s41531-023-00459-3 36739293 PMC9899244

[26] McPherson P. S. A presynaptic inositol-5-phosphatase. Nature 379, 353–357 (1996). 10.1038/379353a0 8552192

[27] Choudhry H., Aggarwal M. & Pan P. Y. Mini-review: Synaptojanin 1 and its implications in membrane trafficking. Neurosci Lett 765, 136288 (2021). 10.1016/j.neulet.2021.136288 34637856 PMC8572151

[28] Vanhauwaert R. The SAC1 domain in synaptojanin is required for autophagosome maturation at presynaptic terminals. EMBO J 36, 1392–1411 (2017). 10.15252/embj.201695773 28331029 PMC5430236

[29] Posor Y., Jang W. & Haucke V. Phosphoinositides as membrane organizers. Nat Rev Mol Cell Biol (2022). 10.1038/s41580-022-00490-x PMC9117997 35589852

[30] Arumugam S. & Kaur A. The Lipids of the Early Endosomes: Making Multimodality Work. Chembiochem 18, 1053–1060 (2017). 10.1002/cbic.201700046 28374483

[31] Chandra M., Kendall A. K., Ford M. G. J. & Jackson L. P. VARP binds SNX27 to promote endosomal supercomplex formation on membranes. Sci Adv 11, eadr9340 (2025). 10.1126/sciadv.adr9340 39937906 PMC11817943

[32] Cossec J. C. Trisomy for synaptojanin1 in Down syndrome is functionally linked to the enlargement of early endosomes. Hum Mol Genet 21, 3156–3172 (2012). 10.1093/hmg/dds142 22511594 PMC3384382

[33] Fasano D. Alteration of endosomal trafficking is associated with early-onset parkinsonism caused by SYNJ1 mutations. Cell Death Dis 9, 385 (2018). 10.1038/s41419-018-0410-7 29515184 PMC5841278

[34] Pan P. Y. Synaptojanin1 deficiency upregulates basal autophagosome formation in astrocytes. J Biol Chem 297, 100873 (2021). 10.1016/j.jbc.2021.100873 34126070 PMC8258991

[35] Coukos R. & Krainc D. Key genes and convergent pathogenic mechanisms in Parkinson disease. Nat Rev Neurosci 25, 393–413 (2024). 10.1038/s41583-024-00812-2 38600347

[36] Abeliovich A. & Gitler A. D. Defects in trafficking bridge Parkinson’s disease pathology and genetics. Nature 539, 207–216 (2016). 10.1038/nature20414 27830778

[37] Pan P. Y., Zhu Y., Shen Y. & Yue Z. Crosstalk between presynaptic trafficking and autophagy in Parkinson’s disease. Neurobiol Dis 122, 64–71 (2019). 10.1016/j.nbd.2018.04.020 29723605 PMC10942671

[38] Wong Y. C. Neuronal vulnerability in Parkinson disease: Should the focus be on axons and synaptic terminals? Mov Disord 34, 1406–1422 (2019). 10.1002/mds.27823 31483900 PMC6879792

[39] Kearney P. J. Silencing Parkinson’s risk allele Rit2 sex-specifically compromises motor function and dopamine neuron viability. NPJ Parkinsons Dis 10, 41 (2024). 10.1038/s41531-024-00648-8 38395968 PMC10891080

[40] Cremona O. Essential role of phosphoinositide metabolism in synaptic vesicle recycling. Cell 99, 179–188 (1999). 10.1016/s0092-8674(00)81649-9 10535736

[41] Luthi A. Synaptojanin 1 contributes to maintaining the stability of GABAergic transmission in primary cultures of cortical neurons. J Neurosci 21, 9101–9111 (2001). 10.1523/JNEUROSCI.21-23-09101.2001 11717343 PMC6763888

[42] Rothstein J. D. Knockout of glutamate transporters reveals a major role for astroglial transport in excitotoxicity and clearance of glutamate. Neuron 16, 675–686 (1996). 10.1016/s0896-6273(00)80086-0 8785064

[43] Jones S. R. Profound neuronal plasticity in response to inactivation of the dopamine transporter. Proc Natl Acad Sci U S A 95, 4029–4034 (1998). 10.1073/pnas.95.7.4029 9520487 PMC19957

[44] Ford C. P. The role of D2-autoreceptors in regulating dopamine neuron activity and transmission. Neuroscience 282, 13–22 (2014). 10.1016/j.neuroscience.2014.01.025 24463000 PMC4108583

[45] Neve K. A., Seamans J. K. & Trantham-Davidson H. Dopamine receptor signaling. J Recept Signal Transduct Res 24, 165–205 (2004). 10.1081/rrs-200029981 15521361

[46] Jeong S. W. & Ikeda S. R. Sequestration of G-protein beta gamma subunits by different G-protein alpha subunits blocks voltage-dependent modulation of Ca2+ channels in rat sympathetic neurons. J Neurosci 19, 4755–4761 (1999). 10.1523/JNEUROSCI.19-12-04755.1999 10366609 PMC6782646

[47] Herlitze S. Modulation of Ca2+ channels by G-protein beta gamma subunits. Nature 380, 258–262 (1996). 10.1038/380258a0 8637576

[48] Phillips P. E. & Stamford J. A. Differential recruitment of N-, P- and Q-type voltage-operated calcium channels in striatal dopamine release evoked by ‘regular’ and ‘burst’ firing. Brain Res 884, 139–146 (2000). 10.1016/s0006-8993(00)02958-9 11082495

[49] Onali P. & Olianas M. C. Involvement of adenylate cyclase inhibition in dopamine autoreceptor regulation of tyrosine hydroxylase in rat nucleus accumbens. Neurosci Lett 102, 91–96 (1989). 10.1016/0304-3940(89)90313-3 2571111

[50] O’Hara C. M., Uhland-Smith A., O’Malley K. L. & Todd R. D. Inhibition of dopamine synthesis by dopamine D2 and D3 but not D4 receptors. J Pharmacol Exp Ther 277, 186–192 (1996). 8613917

[51] Anzalone A. Dual control of dopamine synthesis and release by presynaptic and postsynaptic dopamine D2 receptors. J Neurosci 32, 9023–9034 (2012). 10.1523/JNEUROSCI.0918-12.2012 22745501 PMC3752062

[52] Mayfield R. D. & Zahniser N. R. Dopamine D2 receptor regulation of the dopamine transporter expressed in Xenopus laevis oocytes is voltage-independent. Mol Pharmacol 59, 113–121 (2001). 10.1124/mol.59.1.113 11125031

[53] Lycas M. D. Nanoscopic dopamine transporter distribution and conformation are inversely regulated by excitatory drive and D2 autoreceptor activity. Cell Rep 40, 111431 (2022). 10.1016/j.celrep.2022.111431 36170827 PMC9617621

[54] Lee F. J. Dopamine transporter cell surface localization facilitated by a direct interaction with the dopamine D2 receptor. EMBO J 26, 2127–2136 (2007). 10.1038/sj.emboj.7601656 17380124 PMC1852782

[55] Kearney P. J. Presynaptic Gq-coupled receptors drive biphasic dopamine transporter trafficking that modulates dopamine clearance and motor function. J Biol Chem 299, 102900 (2023). 10.1016/j.jbc.2023.102900 36640864 PMC9943899

[56] Wu S. The Dopamine Transporter Recycles via a Retromer-Dependent Postendocytic Mechanism: Tracking Studies Using a Novel Fluorophore-Coupling Approach. J Neurosci 37, 9438–9452 (2017). 10.1523/JNEUROSCI.3885-16.2017 28847807 PMC5618262

[57] Wheeler D. S. Amphetamine activates Rho GTPase signaling to mediate dopamine transporter internalization and acute behavioral effects of amphetamine. Proc Natl Acad Sci U S A 112, E7138–7147 (2015). 10.1073/pnas.1511670112 26553986 PMC4697400

[58] Holton K. L., Loder M. K. & Melikian H. E. Nonclassical, distinct endocytic signals dictate constitutive and PKC-regulated neurotransmitter transporter internalization. Nat Neurosci 8, 881–888 (2005). 10.1038/nn1478 15924135 PMC2597780

[59] Sorkina T., Hoover B. R., Zahniser N. R. & Sorkin A. Constitutive and protein kinase C-induced internalization of the dopamine transporter is mediated by a clathrin-dependent mechanism. Traffic 6, 157–170 (2005). 10.1111/j.1600-0854.2005.00259.x 15634215

[60] Hoover B. R., Everett C. V., Sorkin A. & Zahniser N. R. Rapid regulation of dopamine transporters by tyrosine kinases in rat neuronal preparations. J Neurochem 101, 1258–1271 (2007). 10.1111/j.1471-4159.2007.04522.x 17419806

[61] Kaasinen V., Vahlberg T., Stoessl A. J., Strafella A. P. & Antonini A. Dopamine Receptors in Parkinson’s Disease: A Meta-Analysis of Imaging Studies. Mov Disord 36, 1781–1791 (2021). 10.1002/mds.28632 33955044

[62] Niccolini F., Su P. & Politis M. Dopamine receptor mapping with PET imaging in Parkinson’s disease. J Neurol 261, 2251–2263 (2014). 10.1007/s00415-014-7302-2 24627109

[63] Saez M. D2 dopamine receptors and the striatopallidal pathway modulate L-DOPA-induced dyskinesia in the mouse. Neurobiol Dis 186, 106278 (2023). 10.1016/j.nbd.2023.106278 37683958

[64] Saenz J. Parkinson’s disease gene, Synaptojanin1, dysregulates the surface maintenance of the dopamine transporter. NPJ Parkinsons Dis 10, 148 (2024). 10.1038/s41531-024-00769-0 39117637 PMC11310474

[65] Lin M. In Parkinson’s patient-derived dopamine neurons, the triplication of alpha-synuclein locus induces distinctive firing pattern by impeding D2 receptor autoinhibition. Acta Neuropathol Commun 9, 107 (2021). 10.1186/s40478-021-01203-9 34099060 PMC8185945

[66] Liu A. pHmScarlet is a pH-sensitive red fluorescent protein to monitor exocytosis docking and fusion steps. Nat Commun 12, 1413 (2021). 10.1038/s41467-021-21666-7 33658493 PMC7930027

[67] Saenz J. Parkinson’s disease gene, Synaptojanin1, dysregulates the surface maintenance of the dopamine transporter. Npj Parkinsons Disease 10 (2024). 10.1038/s41531-024-00769-0 PMC11310474 39117637

[68] Klein Herenbrink C. Multimodal detection of dopamine by sniffer cells expressing genetically encoded fluorescent sensors. Commun Biol 5, 578 (2022). 10.1038/s42003-022-03488-5 35689020 PMC9187629

[69] Dagra A. alpha-Synuclein-induced dysregulation of neuronal activity contributes to murine dopamine neuron vulnerability. NPJ Parkinsons Dis 7, 76 (2021). 10.1038/s41531-021-00210-w 34408150 PMC8373893

[70] Meredith G. E. Immunocytochemical characterization of catecholaminergic neurons in the rat striatum following dopamine-depleting lesions. Eur J Neurosci 11, 3585–3596 (1999). 10.1046/j.1460-9568.1999.00774.x 10564366

[71] Mazloom M. & Smith Y. Synaptic microcircuitry of tyrosine hydroxylase-containing neurons and terminals in the striatum of 1-methyl-4-phenyl-1,2,3,6-tetrahydropyridine-treated monkeys. J Comp Neurol 495, 453–469 (2006). 10.1002/cne.20894 16485290 PMC2597082

[72] Cao M., Park D., Wu Y. & De Camilli P. Absence of Sac2/INPP5F enhances the phenotype of a Parkinson’s disease mutation of synaptojanin 1. Proc Natl Acad Sci U S A 117, 12428–12434 (2020). 10.1073/pnas.2004335117 32424101 PMC7275725

[73] Priya A., Kalaidzidis I. V., Kalaidzidis Y., Lambright D. & Datta S. Molecular insights into Rab7-mediated endosomal recruitment of core retromer: deciphering the role of Vps26 and Vps35. Traffic 16, 68–84 (2015). 10.1111/tra.12237 25367362

[74] Harrison M. S. A mechanism for retromer endosomal coat complex assembly with cargo. Proc Natl Acad Sci U S A 111, 267–272 (2014). 10.1073/pnas.1316482111 24344282 PMC3890810

[75] Sapmaz A. USP32 regulates late endosomal transport and recycling through deubiquitylation of Rab7. Nat Commun 10, 1454 (2019). 10.1038/s41467-019-09437-x 30926795 PMC6440979

[76] Ketel K. A phosphoinositide conversion mechanism for exit from endosomes. Nature 529, 408–412 (2016). 10.1038/nature16516 26760201

[77] Jani R. A. PI4P and BLOC-1 remodel endosomal membranes into tubules. J Cell Biol 221 (2022). 10.1083/jcb.202110132 PMC9524204 36169638

[78] Nitzsche A. Paladin is a phosphoinositide phosphatase regulating endosomal VEGFR2 signalling and angiogenesis. EMBO Rep 22, e50218 (2021). 10.15252/embr.202050218 33369848 PMC7857541

[79] Mani M. The dual phosphatase activity of synaptojanin1 is required for both efficient synaptic vesicle endocytosis and reavailability at nerve terminals. Neuron 56, 1004–1018 (2007). 10.1016/j.neuron.2007.10.032 18093523 PMC3653591

[80] Harris T. W., Hartwieg E., Horvitz H. R. & Jorgensen E. M. Mutations in synaptojanin disrupt synaptic vesicle recycling. J Cell Biol 150, 589–600 (2000). 10.1083/jcb.150.3.589 10931870 PMC2175188

[81] McPherson P. S., Takei K., Schmid S. L. & De Camilli P. p145, a major Grb2-binding protein in brain, is co-localized with dynamin in nerve terminals where it undergoes activity-dependent dephosphorylation. J Biol Chem 269, 30132–30139 (1994). 7982917

[82] Pan P. Y. Parkinson’s Disease-Associated LRRK2 Hyperactive Kinase Mutant Disrupts Synaptic Vesicle Trafficking in Ventral Midbrain Neurons. J Neurosci 37, 11366–11376 (2017). 10.1523/JNEUROSCI.0964-17.2017 29054882 PMC5700420

[83] Hundley F. V. Endo-IP and lyso-IP toolkit for endolysosomal profiling of human-induced neurons. Proc Natl Acad Sci U S A 121, e2419079121 (2024). 10.1073/pnas.2419079121 39636867 PMC11670117

[84] Baba T., Toth D. J., Sengupta N., Kim Y. J. & Balla T. Phosphatidylinositol 4,5-bisphosphate controls Rab7 and PLEKHM1 membrane cycling during autophagosome-lysosome fusion. EMBO J 38, e100312 (2019). 10.15252/embj.2018100312 31368593 PMC6463214

[85] Zhu X., Prakash S. S., McAuliffe G. & Pan P. Y. Synaptojanin1 Modifies Endolysosomal Parameters in Cultured Ventral Midbrain Neurons. eNeuro 10 (2023). 10.1523/ENEURO.0426-22.2023 PMC10166127 37072173

[86] Cullen P. J. & Korswagen H. C. Sorting nexins provide diversity for retromer-dependent trafficking events. Nat Cell Biol 14, 29–37 (2011). 10.1038/ncb2374 22193161 PMC3613977

[87] Hsu F., Hu F. & Mao Y. Spatiotemporal control of phosphatidylinositol 4-phosphate by Sac2 regulates endocytic recycling. J Cell Biol 209, 97–110 (2015). 10.1083/jcb.201408027 25869669 PMC4395482

[88] Jeyasimman D. PDZD-8 and TEX-2 regulate endosomal PI(4,5)P(2) homeostasis via lipid transport to promote embryogenesis in C. elegans. Nat Commun 12, 6065 (2021). 10.1038/s41467-021-26177-z 34663803 PMC8523718

[89] Sun M., Luong G., Plastikwala F. & Sun Y. Control of Rab7a activity and localization through endosomal type Igamma PIP 5-kinase is required for endosome maturation and lysosome function. FASEB J 34, 2730–2748 (2020). 10.1096/fj.201901830R 31908013 PMC7018547

[90] Cao M. Parkinson Sac Domain Mutation in Synaptojanin 1 Impairs Clathrin Uncoating at Synapses and Triggers Dystrophic Changes in Dopaminergic Axons. Neuron 93, 882–896 e885 (2017). 10.1016/j.neuron.2017.01.019 28231468 PMC5340420

[91] Muzio L. Retromer stabilization results in neuroprotection in a model of Amyotrophic Lateral Sclerosis. Nat Commun 11, 3848 (2020). 10.1038/s41467-020-17524-7 32737286 PMC7395176

[92] Eleuteri S. & Albanese A. VPS35-Based Approach: A Potential Innovative Treatment in Parkinson’s Disease. Front Neurol 10, 1272 (2019). 10.3389/fneur.2019.01272 31920908 PMC6928206

[93] Saenz J. Cocaine-regulated trafficking of dopamine transporters in cultured neurons revealed by a pH sensitive reporter. iScience 26, 105782 (2023). 10.1016/j.isci.2022.105782 36594015 PMC9804146

[94] Khezerlou E., Saenz J., Prakash S. S. & Pan P. Y. Protocol for live neuron imaging analysis of basal surface fraction and dynamic availability of the dopamine transporter using DAT-pHluorin. STAR Protoc 5, 103358 (2024). 10.1016/j.xpro.2024.103358 39368094 PMC11490699

[95] Pan P. Y. Synj1 haploinsufficiency causes dopamine neuron vulnerability and alpha-synuclein accumulation in mice. Hum Mol Genet 29, 2300–2312 (2020). 10.1093/hmg/ddaa080 32356558 PMC7424763

[96] Cornejo V. H. Non-conventional Axonal Organelles Control TRPM8 Ion Channel Trafficking and Peripheral Cold Sensing. Cell Rep 30, 4505–4517 e4505 (2020). 10.1016/j.celrep.2020.03.017 32234483

[97] Ramirez O., Garcia A., Rojas R., Couve A. & Hartel S. Confined displacement algorithm determines true and random colocalization in fluorescence microscopy. J Microsc 239, 173–183 (2010). 10.1111/j.1365-2818.2010.03369.x 20701655

[98] Lim A., Rechtsteiner A. & Saxton W. M. Two kinesins drive anterograde neuropeptide transport. Mol Biol Cell 28, 3542–3553 (2017). 10.1091/mbc.E16-12-0820 28904207 PMC5683764

[99] Guo Z., Tong C., Yang Y. & Liu J. J. Immunofluorescence staining of phosphoinositides in primary mouse hippocampal neurons in dissociated culture. STAR Protoc 3, 101549 (2022). 10.1016/j.xpro.2022.101549 35842867 PMC9294264

[100] Asano S. M. Expansion Microscopy: Protocols for Imaging Proteins and RNA in Cells and Tissues. Curr Protoc Cell Biol 80, e56 (2018). 10.1002/cpcb.56 30070431 PMC6158110

