# Supplementary material for: Synaptojanin1 regulates synaptic dopamine release and axonal integrity via retromer-dependent endosomal sorting
Source: Res Sq. 2026 Jun 18:rs.3.rs-9695405. Preprint. [Version 1] doi: 10.21203/rs.3.rs-9695405/v1 (PMC13308361; doi:10.21203/rs.3.rs-9695405/v1)
Supplement: 1 [file NIHPPRS9695405V1-supplement-1.pdf]

Supplemental Figures

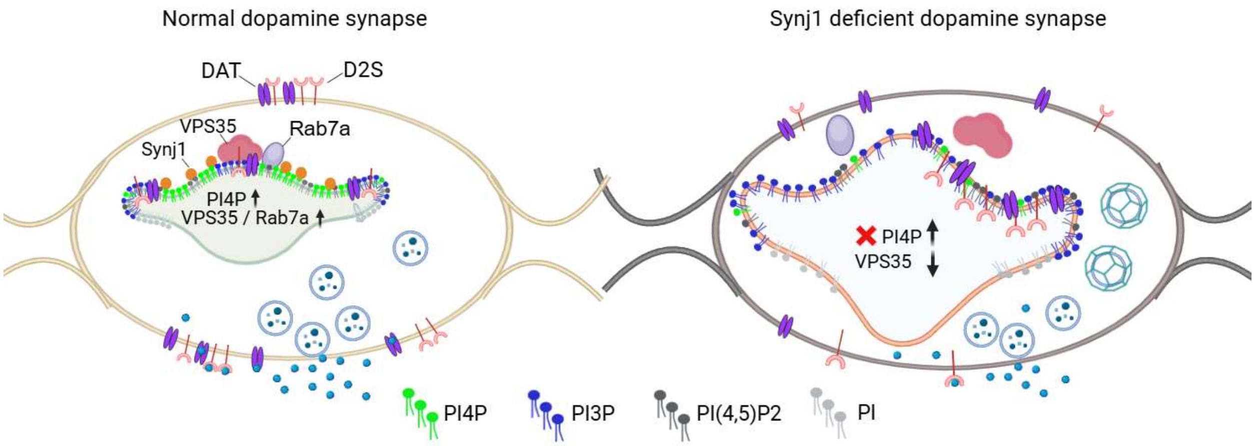

Figure S1: Model of Synj1-VPS35-dependent presynaptic endosomal sorting in dopamine neurotransmission. In dopaminergic neurons, Synj1-dependent control of endosomal PI4P promotes recruitment of VPS35 and Rab7a to presynaptic endosomes, thereby facilitating retromer-mediated sorting and recycling of receptor and transporter cargo to the plasma membrane. This Synj1-mediated endosomal signalling maintains dopamine storage and release in control neurons. In Synj1-depleted neurons, dopamine-induced increases in endosomal PI4P fail, resulting in impaired VPS35 and Rab7a recruitment. Consequently, endosomal sorting and surface delivery of cargo are disrupted, leading to intracellular cargo accumulation and endosomal swelling. These changes impair presynaptic dopamine storage, release and dopamine-dependent behavior.

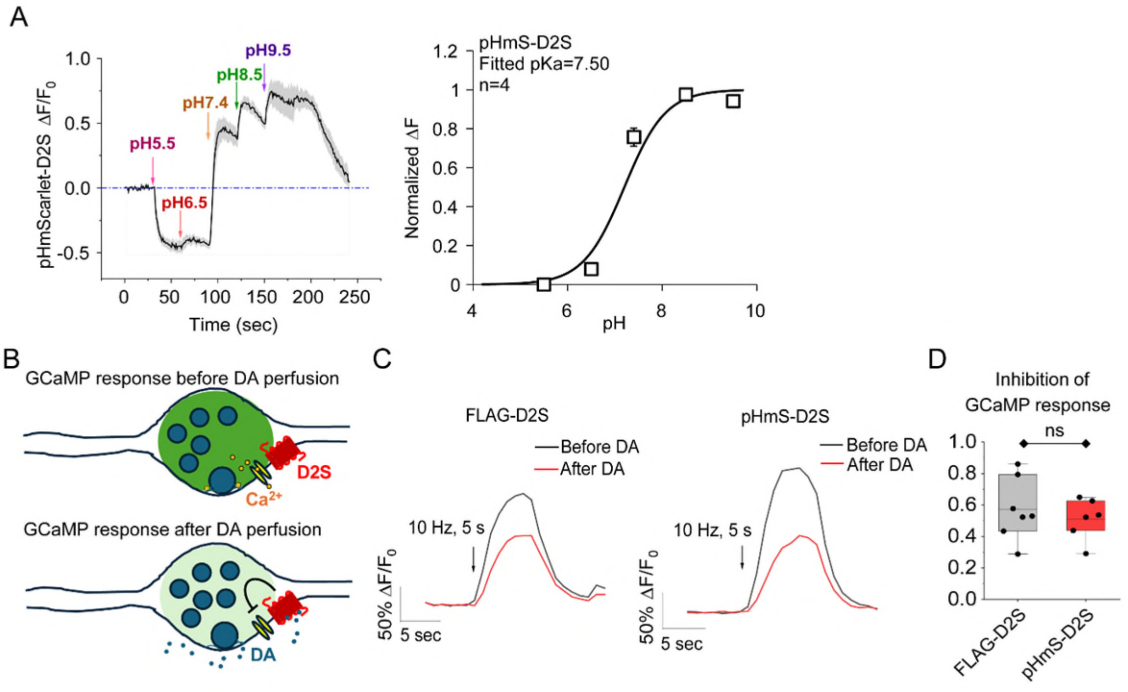

Figure S2: Validation of pHmS-D2S. A) Measurement of the pKa of pHmScarlet-D2S at neuronal axons. Axons expressing pHmScarlet-D2S were sequentially perfused with Tyrode's solutions buffered at defined pH values. The dynamic fluorescence change was rescaled to 1 and the relative DF at each pH was fitted by the Henderson-Hasselbalch equation to find the fitting pka=7.5 (right). Data are presented as mean ± SEM; n=4 independent experiments (left). **B**) Schematic of the assay used to assess tagged D2S function by measuring dopamine (DA)-induced inhibition of presynaptic Ca^{2+}. (**C-D**) Representative GCaMP traces from boutons co-expressing FLAG-D2S or pHmScarlet-D2S (**C**) and quantification of DA-induced inhibition (**D**). ns, non-significant

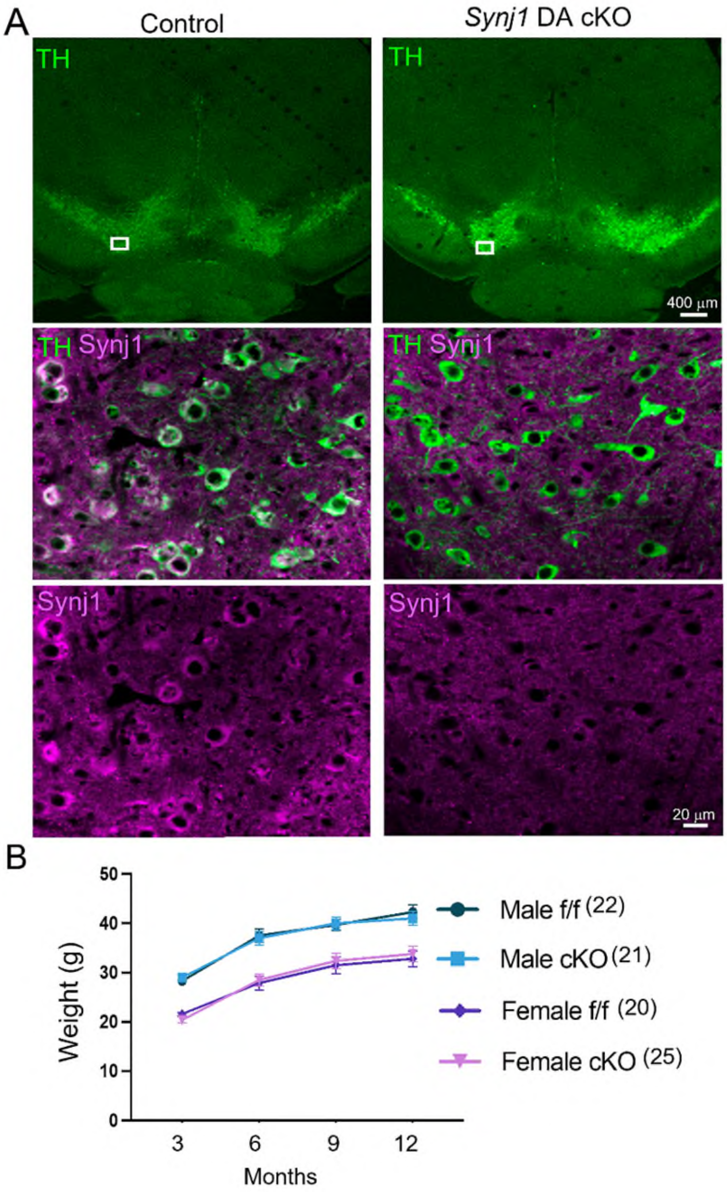

Figure S3: Validation of Synj1 deletion and body weight in *Synj1* DA cKO mice. A) Representative 20× immunofluorescence images of midbrain slices from control (*Synj1*$^{flox/flox}$) and *Synj1* DA cKO mice showing TH and Synj1 staining. Merged images show loss of Synj1 signal in TH-positive neuronal soma in *Synj1* DA cKO mice. Scale bar, 20 µm. **B)** Body weight of control and *Synj1* DA cKO mice measured at 3, 6, 9 and 12 months of age. Both genotypes show a similar age-dependent increase in body weight, with no differences between control and *Synj1* DA cKO mice.

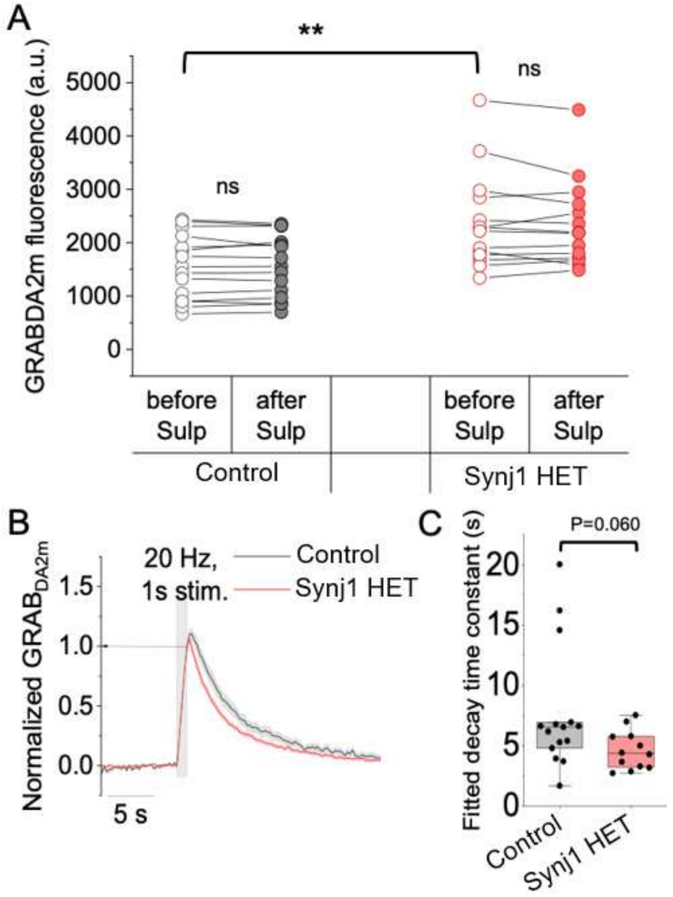

Figure S4: Baseline $GRAB_{DA2m}$ responses in control and *Synj1* HET neurons. A) Baseline GRABDA2m fluorescence in control (*Synj1*$^{+/+}$, n=18) and *Synj1* HET (*Synj1*$^{+/-}$, n=17) neurons before and after 2 min sulpiride treatment. **B)** GRABDA2m fluorescence responses normalized to the fluorescence at the end of stimulation. **C)** Quantification of the decay time constant of GRABDA2m fluorescence in neurons responding to sulpiride in control and *Synj1* HET neurons. Data are presented as mean ± SEM with individual data points shown. Data are from five independent co-culture preparations. Paired two-tailed Student's *t*-test (A) or Mann-Whitney test (C). ns, not significant; **p< 0.001

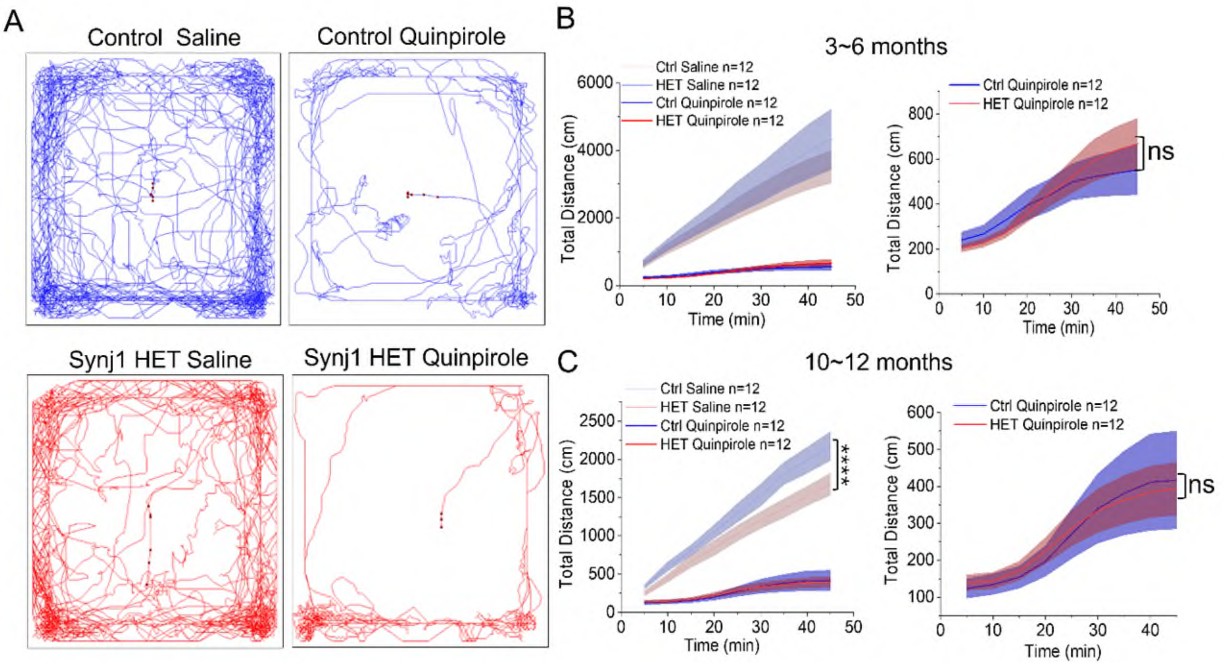

Figure S5: *Synj1* HET female mice do not show impaired D2 receptor pharmacosensitivity. A, B) Locomotor response to the D2-like agonist quinpirole in control (*Synj1*$^{+/+}$) and *Synj1* HET female mice (3-6 months and 10-12 months). Mice received intraperitoneal injections of saline or quinpirole (0.5 mg/kg), and locomotor activity (total distance traveled) was recorded over 45 min in an open field. **B-C)** Cumulative distance traveled under saline and quinpirole conditions. Quinpirole-treated groups are plotted on a separate scale. ns, not significant, ****$p < 0.0001$

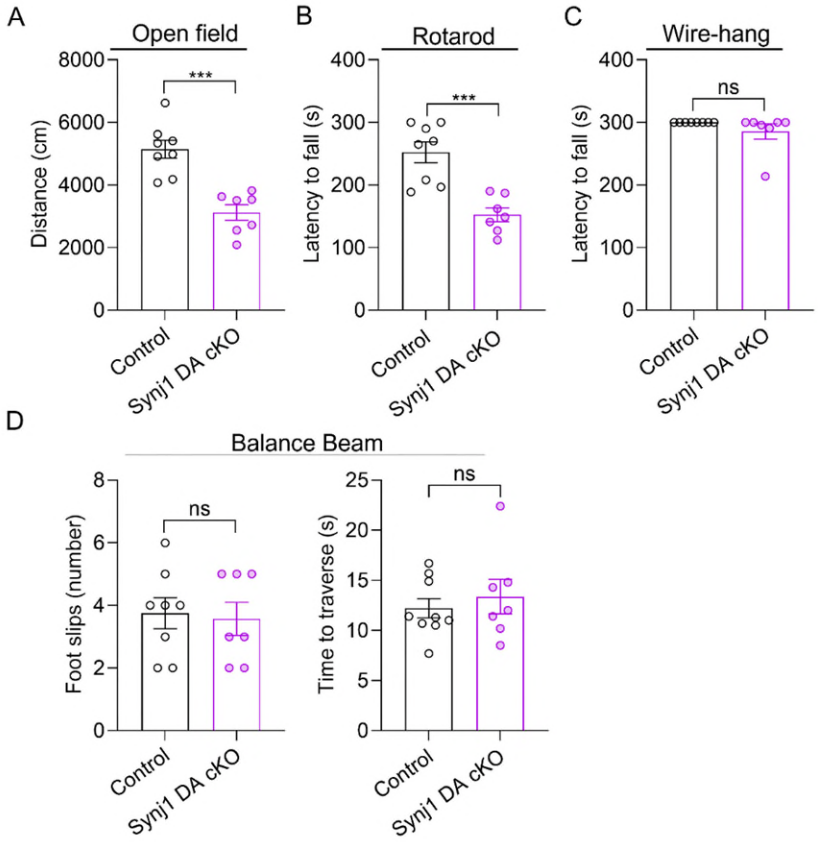

Figure S6: Early motor phenotypes in *Synj1* DA cKO mice (1-2 months). Control (*Synj1*$^{flox/flox}$) and *Synj1* DA cKO mice (male and female, 1-2 months) were subjected to behavioral testing. **A)** Open field analysis showing reduced locomotor activity in *Synj1* DA cKO mice, measured as total distance traveled. **B)** Accelerated rotarod performance showing impaired motor coordination in *Synj1* DA cKO mice. **C)** Wire hang test showing no difference in motor strength in *Synj1* DA cKO mice. **D)** Balance beam test showing no difference in foot slips or traversal time between *Synj1* DA cKO and control mice. ns, not significant, ***$p < 0.001$

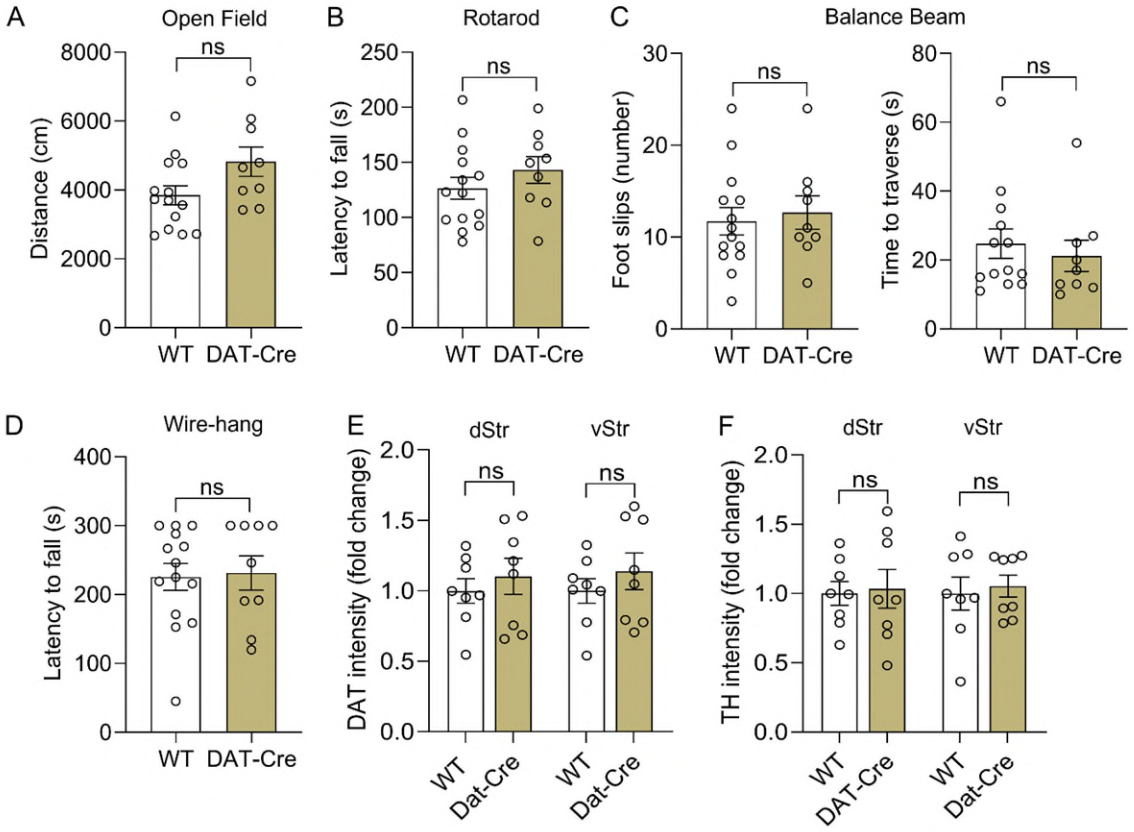

Figure S7: Motor behavior and DAT/ TH expression are unchanged in WT and *DAT-Cre* mice (3–6 month). WT and *DAT-Cre* (+/-) mice (male and female, 3-6 months) were subjected to behavioral testing and histological analysis. **A)** Open field analysis showing no difference in locomotor activity between WT and DAT-Cre mice, measured as total distance traveled. **B)** Accelerated rotarod performance showing no difference in motor coordination between WT and *DAT-Cre* mice. **C)** Wire hang test showing no difference in motor strength between WT and *DAT-Cre* mice. **D)** Balance beam test showing no difference in foot slips or traversal time between WT and DAT-Cre mice. **E)** Quantification of TH and DAT fluorescence intensity in dorsal and ventral striatum from WT and DAT-Cre mice. ns, not significant.

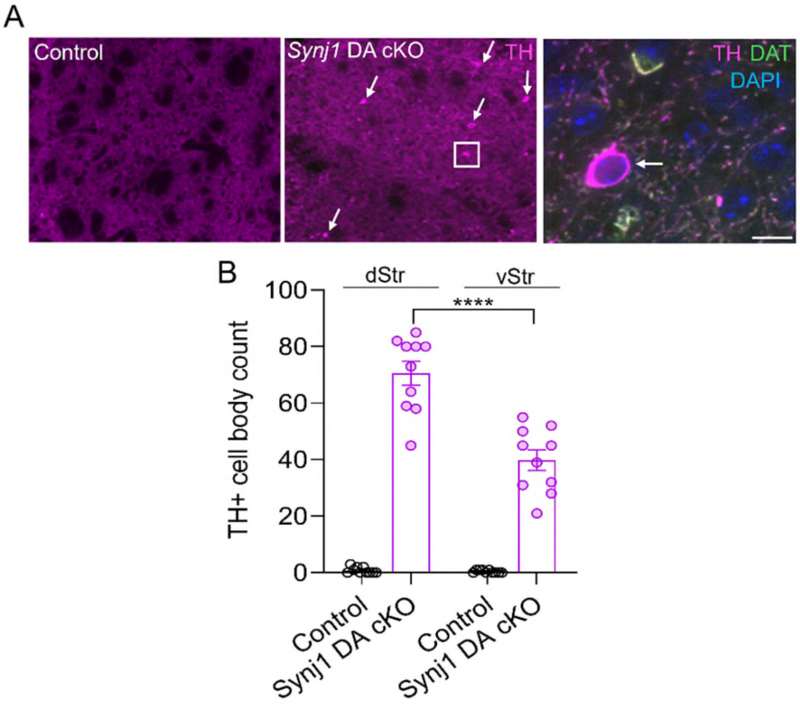

Figure S8: TH-positive cell bodies in the striatum of *Synj1* DA cKO mice. A) Representative images of TH and DAPI staining in striatum sections from control and *Synj1* DA cKO mice. **B)** TH+ cell body count in dorsal and ventral hemi striatum of control and *Synj1* DA cKO mice. Data are shown as mean ± SEM with individual data points. Each symbol is a brain. Statistical significance was determined using unpaired two-tailed Student's *t*-tests. ****$p < 0.0001$

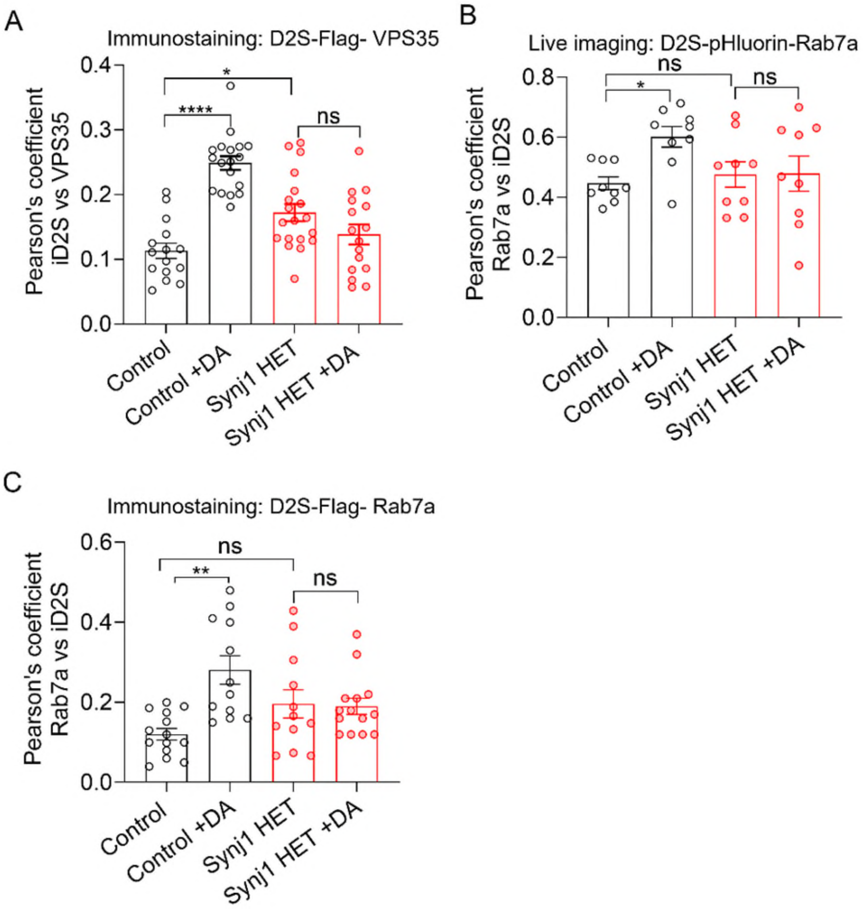

Figure S9: Pearson's correlation coefficient analysis of intracellular D2S colocalization with VPS35 and Rab7a in midbrain neurons. Quantification of colocalization corresponding to Fig.5 using Pearson's correlation coefficient. **A)** Pearson's correlation coefficient analysis of intracellular D2S (iD2S) localization with VPS35 in ventral midbrain control (WT) and *Synj1* HET neurons expressing FLAG-D2S following repeatedly vehicle or 10 µM dopamine (DA) perfusion. **B)** Pearson's coefficient analysis from live imaging of axons expressing pHluorin-D2S and Rab7a across conditions. Neurons co-expressing pHluorin-D2S and TdTomato-Rab7a were analyzed following NH_4Cl perfusion**. C)** Peason's coefficient analysis of endogenous Rab7a with intracellular FLAG-D2S in control and *Synj1* HET midbrain neurons. Neurons were perfused with vehicle or 10 µM DA, followed by surface FLAG blocking and immunolabeling for Rab7a and FLAG. Data are presented as mean ± SEM with individual data points shown. Each data point represents analysis of axons within one field of view. Data are from two independent culture preparations. Statistical significance was determined using two-way ANOVA with Tukey's multiple comparisons test. ns, not significant; $*p < 0.05$, $**p < 0.01$, $****p < 0.0001$

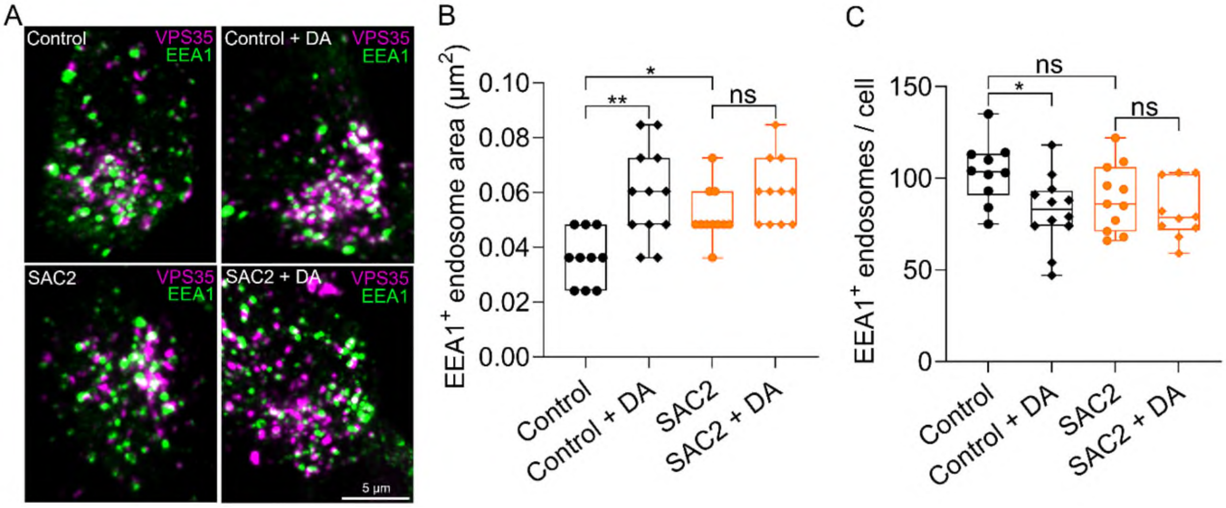

Figure S10: Dopamine increases VPS35 enrichment on EEA1-positive endosomes in control but not in SAC2-expression N2A cells. A) Representative images corresponding to Fig .5L showing VPS35 enrichment on EEA1-positive endosomes in N2A cells co-expressing FLAG-D2S with GFP (control) or SAC2-GFP under basal conditions or following 10 µM dopamine (DA) perfusion. **B, C)** Quantification of EEA1-positive endosomes area (B) and number (C) per cell across conditions. Data are presented as mean ± SEM with individual data points shown. Data from two independent experiments. Statistical analysis was determined using two-way ANOVA with Tukey's multiple comparisons test. ns = non-significant; *$p<0.05$, **$p<0.01$

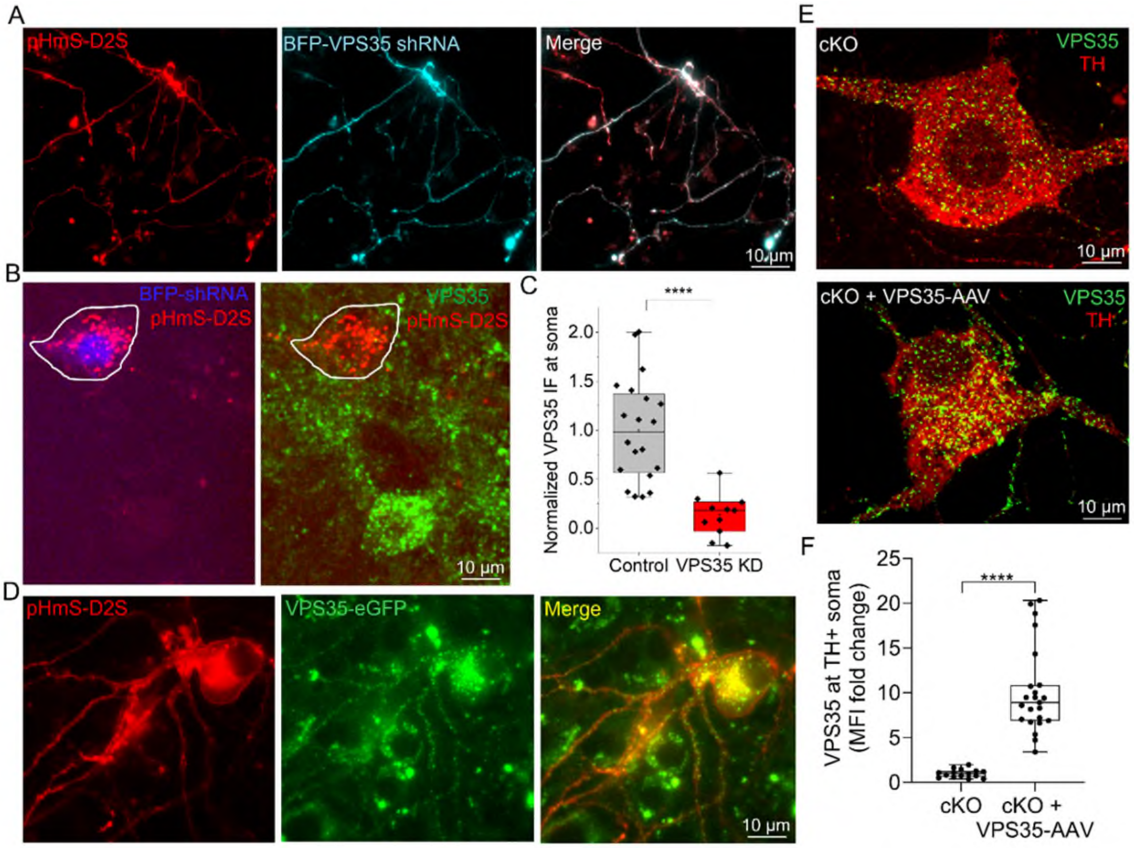

Figure S11: Validation of VPS35 knockdown and overexpression in cultured neurons. A) Live confocal images of ventral midbrain neurons co-expressing pHmScarlet-D2S and BFP-VPS35 shRNA. **B)** VPS35 Immunofluorescence images of neurons expressing pHmScarlet-D2S and BFP-VPS35 shRNA to assess knockdown efficiency. **C)** Quantification of normalized VPS35 fluorescence intensity in neuronal soma following VPS35 shRNA expression. **D)** Live confocal images of ventral midbrain neurons co-expressing pHms-D2S and VPS35-eGFP. **E)** VPS35 immunofluorescence images of *Synj1* DA cKO neurons with or without AAV-DIO-VPS35 expression to assess overexpression efficiency. **F)** Quantification of VPS35 fluorescence intensity at TH+ soma following AAV-DIO-VPS35 expression. Unpaired two-tailed Student's *t*-test. ****$p<0.0001$
